# The influence of substituents in governing the strength of the P–X bonds of substituted halophosphines R^1^R^2^P–X (X = F and Cl)

**DOI:** 10.3389/fchem.2023.1283418

**Published:** 2023-10-03

**Authors:** Robert J. O’Reilly, Amir Karton

**Affiliations:** School of Science and Technology, University of New England, Armidale, NSW, Australia

**Keywords:** chlorophosphine, fluorophosphine, CCSD(T), W2 theory, density functional theory, bond dissociation energy

## Abstract

In this study, the gas-phase homolytic P–F and P–Cl bond dissociation energies (BDEs) of a set of thirty fluorophosphine (R^1^R^2^P–F) and thirty chlorophosphine-type (R^1^R^2^P–Cl) molecules have been obtained using the high-level W2 thermochemical protocol. For the R^1^R^2^P–F species, the P–F BDEs (at 298 K) differ by up to 117.0 kJ mol^−1^, with (H_3_Si)_2_P–F having the lowest BDE (439.5 kJ mol^−1^) and F_2_P–F having the largest BDE (556.5 kJ mol^−1^). In the case of the chlorophosphine-type molecules, the difference in BDEs is considerably smaller (*i.e.*, 72.6 kJ mol^−1^), with (NC)_2_P–Cl having the lowest P–Cl BDE (299.8 kJ mol^−1^) and (HO)_2_P–Cl having the largest (372.4 kJ mol^−1^). We have further analyzed the effect of substituents in governing the P–F and P–Cl BDEs by considering the effect of substituents in the parent halogenated precursors (using molecule stabilization enthalpies) and the effect of substituents in the product radicals (using radical stabilization enthalpies). Finally, we have also assessed the performance of a wide range of DFT methods for their ability to compute the gas-phase P–F and P–Cl BDEs contained in this dataset. We find that, overall, the double hybrid functional DSD-PBEB95 offers the best performance for both bond types, with mean absolute deviations of just 2.1 (P–F BDEs) and 2.2 (P–Cl BDEs) kJ mol^−1^.

## Introduction

Fluorophosphine and chlorophosphine species (*i.e.*, R^1^R^2^P–X, where X = F or Cl) are useful reagents in synthetic organic and transition metal chemistry. Perhaps the most well-studied of these classes of compounds are the trihalides (*i.e.*, PF_3_ and PCl_3_). Both trihalides have been utilized as ligands in transition metal chemistry (Boxhoorn et al., 1979; Davies et al., 1995; Hammill et al., 1997), and using the Quantitative Analysis of Ligand Effects (QALE) model, the comparative properties of PF_3_ and PCl_3_ as ligands have been compared with other phosphorous-based ligands such as PH_3_ and P(CH_2_CH_2_CN)_3_ (Woska et al., 2000). Concerning the molecular geometries of both PF_3_ and PCl_3_, these have been examined previously using high-level quantum chemical calculations (Breidung and Thiel, 2019). Apart from PF_3_ and PCl_3_, the prototypical species, monofluorophosphine (H_2_PF) and monochlorophosphine (H_2_PCl) have both been synthesized, and their IR spectra have been recorded (Beckers, 1993). In addition, difluorophosphine (HPF_2_) has been synthesized (Rudolph and Parry, 1965) and the complexation of this molecule with borane afforded a stable adduct (Rudolph and Parry, 1967).

A number of other phosphorus (III) fluorides have been synthesised including, for example: i) difluoroiodophosphine (F_2_PI) (Rudolph et al., 1966a), which was also used as a substrate by which to produce both μ-oxo-bisdifluorophosphine (F_2_POPF_2_) and cyanodifluorophosphine (F_2_PCN) (Rudolph et al., 1966b), ii) acyclic (McCombie and Saunders, 1946) and cyclic fluorophosphites (Miles-Hobbs et al., 2019; Ibrahim et al., 2022), iii) aminofluorophosphines (Reddy and Schmutzler, 1965), and iv) bis(*t*-butyl)fluorophosphine, the first known stable dialkylfluorophosphine (Stelzer and Schmutzler, 1971). Concerning the chlorinated species (*i.e.*, R^1^R^2^P–Cl), we note that diamino-substituted chlorophosphines, such as bis(*N,N*-diisopropylamino)chlorophosphine (*i.e.*, (^*i*^Pr_2_N)_2_PCl), have been used in: i) the synthesis of phosphoramidites and H-phosphonates of D-nucleosides, as well as facilitating the formation of 3′–5′-internucleotidic phosphonate bonds (Marugg et al., 1986), ii) in the first step of a two-step synthesis of thymidine phosphoramidite monomer building blocks from 5′-*O*-dimethoxytrityl protected thymidine (Zhang et al., 2009), and iii) in a phosphitylation reaction that constituted an early step in the synthesis of 5-ethynylimidazole-4-carboxamide (EICA) nucleotide prodrugs (Nakamura et al., 2022). A number of chlorophosphite derivatives have also seen use in synthetic chemistry. For example,. diethylchlorophosphite (*i.e.*, (EtO)_2_PCl) has been studied in the context of, for example: i) its reaction with triethylphosphite and benzylidenemethylamine, resulting in the formation of diethyl-(*N*-methyl-*N*-α-diethylphosphonebenzyl)amidophosphite (Kibardin et al., 1983), ii) as a reagent in the synthesis of boranucleic acids (Vyakaranam et al., 2001), iii) as a reducing agent for effecting the conversion of nitro compounds to amines (Fischer and Sheihet, 1998), and iv) in facilitating the conversion of aldoximes to nitriles (Jie et al., 2002). Concerning alkyl-substituted species, we note that compounds such as dichloromethylphosphine (MePCl_2_) are used in industrial processes, including the synthesis of Gluphosinate, a herbicide (Svara et al., 2012). We also wish to note that a number of reactions between PCl_3_ and organic molecules have shown to proceed via radical reactions, in which homolytic dissociation of the P–Cl bond is appears to constitute an initial step. These reactions include, for example: i) the formation of dichlorophosphines (*e.g.*, RPCl_2_): by alkylation of PCl_3_ with methane or ethane (Pianfetti and Quin, 1962), and ii) the photochemical radical-based chain reaction between PCl_3_ and alkenes (Little et al., 1966).

Over the past decades, computational chemistry has been extensively used to study the energetic and spectroscopic effects of substituents (Wright et al., 1997; DiLabio et al., 1999; Jacquemin et al., 2005; Chai et al., 2011; Karton et al., 2012; Chan et al., 2016; Sarrami et al., 2017; Rosel and Schreiner, 2022; Vasić et al., 2023). Given the synthetic scope and versatility of fluoro- and chlorophosphine-type molecules, and the potential for reactions between these species and other molecules to proceed via radical-based mechanisms (in which homolytic P–F or P–Cl bond dissociation constitutes a key step) it would be insightful to have a better understanding of the magnitude of the effect of substituents in governing the strength of the P–F and P–Cl bonds of molecules of this type toward homolytic bond dissociation (*i.e.*, to examine how substituents affect the energies associated with the reactions presented in Equations 1, 2).

$$R^{1}R^{2}P–F\;\to \;R^{1}R^{2}P•+F•$$ (1)

$$R^{1}R^{2}P–\text{Cl }\to \;R^{1}R^{2}P•+\text{Cl}•$$ (2)

Having said that, to date, very few studies have investigated the effect of substituents in governing the strength of P–F and P–Cl bonds in trivalent halophosphine-type molecules toward homolytic bond dissociation. To the best of our knowledge, the most comprehensive and accurate set of data reported to date, obtained using the benchmark-quality W1 thermochemical protocol, was that reported by Chan and Radom in their BDE261 dataset (Chan and Radom, 2012). That being said, the number of species was very limited (*i.e.*, they reported P–X BDEs, where X = F and Cl, for H_2_P–X, MeHP–X, Me_2_P–X, FHP–X and F_2_P–X), and so a void still remains concerning the quantitative magnitude regarding the effect of substituents in governing the strength of P–F and P–Cl bonds of trivalent halophosphine-type species toward homolytic dissociation. We note that a recent study also included a subset of P–F and P–Cl BDEs, as part of a more comprehensive dataset of BDEs, which were computed using the ROCBS-QB3 thermochemical protocol without the inclusion of zero-point vibrational energy or enthalpy corrections (Prasad et al., 2021).

In this present study, we use the more rigorous W2 thermochemical protocol to: i) obtain gas-phase P–F and P–Cl BDEs for a wide range of mono- and disubstituted-halophosphines consisting of a diverse set of synthetically-relevant substituents, ii) consider quantitatively how substituents govern the P–F and P–Cl BDEs by inspecting their effects in both the precursor halophosphine-type molecules (*i.e.*, R^1^R^2^P–X) as well as their effects in the product phosphorus-centered radicals (*i.e.*, R^1^R^2^P•), and finally, iii) given the benchmark-quality of the W2 P–F and P–Cl BDEs, use these values in order to assess the performance of a wide range of density functional theory (DFT) and double-hybrid DFT methods (in conjunction with the A′VQZ basis set) for their ability to accurately compute homolytic gas-phase P–X (X = F or Cl) BDEs.

## Computational methods

In order to obtain equilibrium geometries for all of the chloro- and fluorophosphine-type molecules (*i.e.*, R^1^R^2^P–F and R^1^R^2^P–Cl, respectively) as well as the corresponding phosphorous-centered radicals (*i.e.*, R^1^R^2^P•) necessary to compute benchmark-quality P–F and P–Cl BDEs at the W2 level of theory, we employed the B3LYP/A′VTZ level of theory (where A′V*n*Z denotes the use of cc-pV*n*Z basis sets for hydrogen, aug-cc-pV*n*Z for first-row elements, and aug-cc-pV(*n+*d)Z basis sets for second-row elements) (Dunning, 1989; Wilson et al., 1999). Confirmation that each optimized structure corresponded to an equilibrium structure on the potential energy surface was obtained by way of harmonic vibrational frequency calculations (performed at the same level of theory), which demonstrated that all molecules lacked imaginary frequencies.

As alluded to previously, to obtain reliable benchmark-quality P–F and P–Cl BDEs of this set of trivalent halophosphine-type molecules, we have employed the W2 thermochemical protocol (employing geometries, as well as the corresponding zero-point-vibrational-energy (ZPVE) and vibrational corrections to enthalpy (*H*_vib_), which have been obtained at the B3LYP/A′VTZ level). The W2 method constitutes a layered extrapolation to the all-electron relativistic CCSD(T)/CBS (coupled cluster energy with single, double, and quasiperturbative triple excitations at the complete-basis-set limit) (Martin and de Oliveira, 1999; Karton et al., 2006; Karton, 2016; Karton, 2022). In arriving at a final all-electron, relativistic W2 energy, the following steps were performed: i) an underlying SCF/CBS energy was obtained using a two-point extrapolation of the form E(*L*) = E_∞_ + A/L^5^ in conjunction with the A′VQZ and A′V5Z basis sets, ii) a correction for single and double excitations (*i.e.*, ΔCCSD) was obtained using a two-point extrapolation of the form E(L) = E_∞_ + A/L^3^, based on CCSD calculations performed in conjunction with the A′VQZ and A′V5Z basis sets, iii) a correction for parenthetical triples excitations (*i.e.*, Δ(T)) was obtained by way of a two-point extrapolation of the form E(L) = E_∞_ + A/L^3^ in conjunction with the A′VTZ and A′VQZ basis sets, iv) a core-valence correction (ΔCV), which is computed as the difference between the all-electron CCSD(T)/MTsmall energies (with the exception of second-row elements, in which the 1*s* electrons are frozen) and the corresponding frozen core calculations, and v) a scalar relativistic correction (ΔRel), which is obtained by way of second-order Douglass-Kroll-Hess (DKH) calculations (Douglas and Kroll, 1974; Hess, 1985), being computed as the difference in energy between a frozen-core DKH-CCSD(T)/MTsmall and frozen-core CCSD(T)/MTsmall calculation. The bottom-of-the-well non-relativistic all-electron W2 energy (*i.e.*, W2_AE,Rel_) is obtained as the sum of the SCF/CBS, ΔCCSD, Δ(T), ΔCV and ΔRel components. We further wish to note that for all radicals, we have employed the restricted-open-shell (*i.e.*, ROCCSD(T)) formalism.

In order to report P–X BDEs at both 0 K and 298 K, we have additionally corrected our W2_AE,Rel_ energies by adding scaled ZPVEs and *H*_vib_ corrections that were obtained from the harmonic vibrational frequency calculations performed at the B3LYP/A′VTZ level of theory. We have employed scaling factors reported previously in the literature (Merrick et al., 2007) for correcting both the ZPVE (scaled by 0.9884) and *H*_vib_ (scaled by 0.9987) values. Thus, the W2 energy at 0 K (denoted W2_0_) is obtained by adding the scaled ZPVE contribution to the W2_AE,Rel_ energy, while the W2 enthalpy (at 298 K) is obtained by adding the *H*_vib_ correction to the W2_0_ energy.

We have also sought to identify more computationally efficient methods for the computation of homolytic gas-phase P–F and P–Cl BDEs, given that for larger molecules, the use of thermochemical protocols such as W2 may computationally prohibitive. In this regard, we have assessed the performance of a plethora of DFT functionals for their ability to compute gas-phase homolytic P–X (X = F and Cl) BDEs (in conjunction with the A′VQZ basis set). To perform this assessment, we have used as reference values the complete set of W2 non-relativistic bottom-of-the-well P–F and P–Cl BDEs. The DFT exchange-correlation functionals considered in this study, ordered by their rung on Jacob’s Ladder (Perdew and Schmidt, 2001) are the generalized gradient approximation (GGA) functionals: BLYP (Becke, 1988; Lee et al., 1988), B97-D (Grimme, 2006a), HCTH407 (Boese and Handy, 2001) PBE (Perdew et al., 1996), revPBE (Ernzerhof and Perdew, 1998), PB86 (Becke, 1988; Perdew, 1986), and BPW91 (Becke, 1988; Perdew et al., 1992), the meta-GGA functionals: TPSS (Tao et al., 2003), τ-HCTH (Boese and Handy, 2002), VSXC (van Voorhis and Scuseria, 1998), MN12-L (Peverati et al., 2012), MN15-L (Yu et al., 2016), *r* ^2^SCAN (Furness et al., 2020), and B97M-V (Mardirossian and Head-Gordon, 2015); the hybrid-GGAs: BH&HLYP (Becke, 1993a), B3LYP (Lee et al., 1988; Becke, 1993b; Stephens et al., 1994), B3PW91 (Perdew et al., 1992; Becke, 1993b), PBE0 (Adamo and Barone, 1999), B97-1 (Hamprecht et al., 1998), X3LYP (Xu et al., 2005), SOGGA11-X (Peverati and Truhlar, 2011a), APF (Austin et al., 2012), and the range-separated functionals ωB97 (Chai and Head-Gordon, 2008), ωB97X (Chai and Head-Gordon, 2008), N12-SX (Peverati and Truhlar, 2012), CAM-B3LYP (Yanai et al., 2004), ωB97X-V (Mardirossian and Head-Gordon, 2014); the hybrid-meta GGAs (HMGGAs): M06 (Peverati et al., 2012), M06-2X (Zhao and Truhlar, 2008a), M08-HX (Zhao and Truhlar, 2008b), MN15 (Yu et al., 2016), BMK (Boese and Martin, 2004), TPSSh (Staroverov et al., 2003), τ-HCTHh (Boese and Handy, 2002), PW6B95 (Zhao and Truhlar, 2005), and the range separated functionals M11 (Peverati and Truhlar, 2011b), and ωB97M-V (Mardirossian and Head-Gordon, 2006), and the double hybrid (DH) functionals: B2-PLYP (Grimme, 2006b), B2K-PLYP (Tarnopolsky et al., 2008), B2GP-PLYP (Karton et al., 2008), mPW2-PLYP (Schwabe and Grimme, 2006), DSD-PBEP86 (Kozuch and Martin, 2011; Kozuch and Martin, 2013), DSD-BLYP (Kozuch et al., 2010), DSD-PBEB95 (Kozuch and Martin, 2013), PBE0-DH (Brémond and Adamo, 2011), PBEQI-DH (Brémond et al., 2014) and PWPB95 (Goerigk et al., 2011). For twelve functionals, for which Becke-Johnson D3 dispersion corrections are available, we have also assessed the performance of the inclusion of such corrections (with the corrected functionals being assigned with the -D3BJ suffix) (Becke and Johnson, 2005; Grimme et al., 2010; Grimme et al., 2011). All calculations have been performed using the Gaussian 16 program (Revision C.01) (Frisch et al., 2009) and ORCA 5.0 programs (Neese, 2012; Neese et al., 2020).

## Results and discussion

### General overview of the P–F and P–Cl BDEs of halophosphine-type species

In this study, the gas-phase homolytic P–X BDEs of a set of 30 fluorophosphine-type molecules (*i.e.*, R^1^R^2^P–F) and 30 chlorophosphine-type molecules (*i.e.*, R^1^R^2^P–Cl), which result in the formation of either fluorine or chlorine atom and a phosphorous-centered radical (*i.e.*, R^1^R^2^P•), have been computed in conjunction with the high-level W2 theory. We have examined the effect of a diverse range of synthetically-relevant substituents in governing the magnitude of the P–X BDEs. For each molecule, we have tabulated the P–X (X = F and Cl) BDEs at 0 K (BDE_0_) and 298 K (BDE_298_), with the values for the fluorophosphine-type species being reported in Table 1, and those of the chlorophosphine-type species being reported in Table 2. In addition to the BDE_0_ and BDE_298_ values for both the P–F and P–Cl bonds, we have additionally reported the various contributions that lead to these values, namely,: the underlying SCF energies (*i.e.*, ΔSCF), energetic corrections arising as a result singles and doubles excitations (*i.e.*, ΔCCSD), corrections for parenthetical connected triples corrections (*i.e.*, Δ(T)), the core-valence corrections (*i.e.*, ΔCV) and the corrections taking into account scalar relativistic effects (*i.e.*, ΔRel), as well as the contributions for account for the effects of zero-point-vibrational energies (*i.e.*, ΔZPVE) and vibrational enthalpic corrections (*i.e.*, Δ*H*_vib_).

**TABLE 1:** Component breakdown and final W2 gas-phase homolytic P–F bond dissociation energies (in kJ mol^−1^).

System	R^1^	R^2^	ΔSCF	ΔCCSD	Δ(T)	ΔCV	ΔRel	ΔZPVE	BDE_0_ ^a^	Δ*H*_vib_	BDE_298_ ^a^
**F1**	SiH_3_	SiH_3_	294.5	137.7	13.8	0.6	−1.3	−7.5	436.3	3.2	439.5
**F2**	CN	CN	322.7	125.3	10.6	0.2	−1.4	−8.8	447.0	3.3	450.3
**F3**	SiH_3_	H	311.5	136.8	13.4	0.5	−1.4	−10.9	448.4	4.0	452.4
**F4**	CN	H	327.7	129.2	11.6	0.2	−1.4	−12.2	453.5	4.4	458.0
**F5**	HC≡C	H	332.9	126.1	11.1	0.3	−1.4	−12.7	454.7	4.5	459.2
**F6**	PH_2_	PH_2_	317.9	136.0	13.3	0.5	−1.0	−8.5	456.5	3.6	460.1
**F7**	H_2_C=CH	H	342.3	123.0	10.8	0.4	−1.4	−11.7	461.8	3.8	465.6
**F8**	PH_2_	H	328.5	134.3	12.9	0.3	−1.3	−11.5	461.5	4.2	465.8
**F9**	H	H	331.5	135.4	12.9	0.3	−1.5	−15.5	461.5	5.7	467.2
**F10**	CH_3_	SiH_3_	328.5	136.4	13.3	0.6	−1.3	−8.7	467.2	3.7	470.9
**F11**	CHO	H	338.1	131.2	12.2	0.4	−1.5	−10.9	467.9	4.0	471.9
**F12**	BH_2_	H	331.2	137.2	13.7	0.6	−1.7	−11.3	468.0	4.2	472.2
**F13**	SH	H	337.8	131.9	12.4	0.2	−1.4	−11.3	468.1	4.4	472.5
**F14**	SH	SH	338.0	133.6	12.8	0.2	−1.3	−7.8	473.9	3.3	477.3
**F15**	Cl	H	343.8	135.7	13.1	0.2	−1.4	−11.9	477.9	4.6	482.5
**F16**	CH_3_	H	349.7	135.1	12.9	0.4	−1.4	−12.4	482.7	4.6	487.3
**F17**	BH_2_	BH_2_	343.3	140.3	15.0	0.9	−2.0	−10.7	485.2	3.7	488.9
**F18**	Cl	Cl	349.2	136.1	13.3	0.1	−1.3	−8.1	487.7	3.6	491.3
**F19**	CN	NH_2_	365.3	124.8	10.6	0.1	−1.4	−11.5	486.3	5.2	491.5
**F20**	NO_2_	H	353.0	137.3	12.9	0.4	−1.4	−12.7	487.9	4.7	492.7
**F21**	CH_3_	Cl	357.7	136.0	13.2	0.3	−1.3	−9.2	495.1	3.9	498.9
**F22**	CH_3_	CH_3_	365.6	134.9	12.9	0.6	−1.4	−10.1	500.9	4.1	505.0
**F23**	NH_2_	H	373.9	130.7	12.1	0.3	−1.4	−13.9	499.9	5.6	505.6
**F24**	OH	H	375.3	133.0	12.3	0.3	−1.5	−12.6	505.2	4.9	510.1
**F25**	F	H	376.0	135.9	12.8	0.2	−1.5	−12.7	508.9	4.9	513.9
**F26**	NH_2_	Cl	379.3	133.7	12.2	0.1	−1.6	−9.6	512.6	4.1	516.7
**F27**	NH_2_	NH_2_	393.9	131.7	12.1	0.3	−1.4	−10.4	524.6	4.3	528.9
**F28**	CH_3_	F	389.8	135.2	12.7	0.3	−1.5	−10.0	525.0	4.2	529.2
**F29**	OH	OH	419.3	131.1	11.7	0.3	−1.5	−10.4	548.8	4.7	553.5
**F30**	F	F	418.4	134.5	12.1	0.1	−1.6	−9.8	552.2	4.3	556.5

^a^ These values include an atomic spin orbit correction of 1.61 kJ mol^−1^, for fluorine atom, taken from Martin et al. (1999).

**TABLE 2:** Component breakdown and final W2 gas-phase homolytic P–Cl bond dissociation energies (in kJ mol^−1^).

Molecule	R^1^	R^2^	ΔSCF	ΔCCSD	Δ(T)	ΔCV	ΔRel	ΔZPVE	BDE_0_ ^a^	Δ*H*_vib_	BDE_298_ ^a^
**Cl1**	CN	CN	212.2	83.0	12.5	0.4	−1.1	−6.1	297.4	2.4	299.8
**Cl2**	CN	H	224.8	85.0	12.8	0.5	−1.1	−9.5	308.9	3.8	312.7
**Cl3**	HC≡C	H	230.3	81.2	12.2	0.5	−1.1	−10.0	309.6	3.8	313.4
**Cl4**	SH	SH	219.2	88.8	14.7	0.4	−0.7	−4.8	314.0	2.2	316.2
**Cl5**	SH	H	230.7	85.7	13.4	0.4	−1.0	−8.3	317.4	3.5	320.8
**Cl6**	Cl	Cl	219.7	93.3	15.4	0.3	−0.8	−5.0	319.3	2.6	321.8
**Cl7**	PH_2_	PH_2_	223.5	91.0	14.9	0.6	−0.6	−6.4	319.6	2.8	322.4
**Cl8**	SiH_3_	SiH_3_	220.6	93.6	15.4	0.8	−1.0	−5.9	320.0	2.7	322.7
**Cl9**	H_2_C=CH	H	243.2	77.9	11.9	0.5	−1.1	−9.1	319.9	3.0	323.0
**Cl10**	SiH_3_	H	228.0	91.8	14.5	0.7	−1.1	−8.8	321.6	3.5	325.0
**Cl11**	PH_2_	H	233.1	88.4	13.9	0.5	−1.0	−9.0	322.3	3.5	325.8
**Cl12**	CN	NH_2_	241.9	80.7	12.6	0.2	−0.9	−8.9	322.2	4.4	326.6
**Cl13**	Cl	H	232.8	90.7	14.3	0.4	−1.0	−9.0	324.7	3.9	328.5
**Cl14**	H	H	237.9	89.2	13.4	0.5	−1.2	−12.9	323.4	5.2	328.6
**Cl15**	CHO	H	242.1	87.1	13.9	0.6	−1.2	−8.5	330.5	3.2	333.7
**Cl16**	BH_2_	H	240.5	90.6	14.3	0.6	−1.5	−9.2	331.8	3.6	335.4
**Cl17**	CH_3_	SiH_3_	240.8	92.2	14.9	0.7	−1.1	−7.0	336.9	3.1	340.0
**Cl18**	CH_3_	Cl	241.1	92.2	14.9	0.4	−0.9	−6.7	337.5	3.0	340.6
**Cl19**	NO_2_	H	241.6	94.8	15.2	0.6	−1.1	−9.7	337.9	3.9	341.8
**Cl20**	CH_3_	H	251.3	89.5	13.8	0.6	−1.1	−10.1	340.4	4.0	344.4
**Cl21**	NH_2_	Cl	248.4	90.7	14.2	0.3	−1.1	−7.0	342.0	3.4	345.4
**Cl22**	NH_2_	H	263.2	83.0	13.0	0.3	−1.0	−11.4	343.7	4.9	348.6
**Cl23**	BH_2_	BH_2_	249.7	93.7	15.8	0.9	−1.8	−8.2	346.5	2.9	349.4
**Cl24**	F	H	255.4	90.4	13.8	0.4	−1.1	−9.8	345.5	4.2	349.7
**Cl25**	OH	H	260.8	85.2	12.8	0.5	−1.0	−8.8	345.9	4.1	350.0
**Cl26**	CH_3_	CH_3_	262.6	90.2	14.3	0.6	−1.1	−8.2	354.9	3.5	358.4
**Cl27**	NH_2_	NH_2_	268.0	86.1	14.0	0.3	−0.9	−7.7	356.3	3.3	359.6
**Cl28**	CH_3_	F	263.9	90.7	14.2	0.4	−1.1	−7.4	357.3	3.3	360.6
**Cl29**	F	F	268.6	90.2	13.8	0.2	−1.0	−6.5	361.8	3.3	365.1
**Cl30**	OH	OH	281.2	85.5	13.5	0.4	−1.0	−7.5	368.7	3.7	372.4

^a^ These values include an atomic spin orbit correction of 3.52 kJ mol^−1^, for chlorine atom, taken from Martin et al. (1999).

Prior to discussing the effect of substituents in governing the magnitude of the gas-phase homolytic P–F and P–Cl BDEs of this set of halophosphine-type molecules, we first wish to provide clarification concerning: i) whether the use of a single-reference method such as CCSD(T) is appropriate for the computation of reliable P–F and P–Cl BDEs for the molecules considered in this study, and ii) whether post-CCSD(T) corrections, which are not taken into consideration at the W2 level, are likely to result in significant additional contributions to the P–X (X = F and Cl) BDEs of the molecules in this set. Beginning with the question of the validity of the use of a single-reference method (*i.e.*, CCSD(T)) for computing the BDEs of these types of molecules, we have chosen to use the *T*_1_ diagnostic to facilitate our consideration of this point. We note that as a threshold, a *T*_1_ diagnostic of ≤0.02 for a given molecule would tend to indicate that the use of a single reference method is appropriate for adequately describing the electronic structure of that system (Lee and Taylor, 1989). In the context of the molecules in this study, we note that, with the exception of only three radical species (namely (H_2_B)_2_PH•, (H_2_C=CH)PH• and (O_2_N)PH•, for which we compute *T*_1_ diagnostics of 0.03), all other molecules are associated with *T*_1_ diagnostics of ≤0.02. On this basis, apart from the possible exception of the three radicals mentioned above, it would stand to reason that the use of the single-reference CCSD(T) method should be appropriate for computing the P–X BDEs of the molecules in our dataset. We now turn our attention to the second point of consideration, namely, the extent to which post-CCSD(T) corrections are likely to alter the P–F and P–Cl BDEs computed at the CCSD(T)/CBS level. To shed light on this question, we have opted to use the %TAE [(T)] diagnostic (that is, the percentage of the total atomization energy accounted for by the quasi-perturbative triples excitations). It has been shown previously that for molecules in which %TAE [(T)] ≤ 5%, post-CCSD(T) corrections are unlikely to be larger than ∼2 kJ mol^−1^. In the context of the molecules in this study, the largest %TAE [(T)] values are observed in the case of (O_2_N)PHF (5.4%) (O_2_N)PHCl (6.0%) and (O_2_N)PH• (6.4%), with all other molecules having %TAE [(T)] values that are ≤5%. Therefore, except for the nitro-substituted species, in which post-CCSD(T) corrections may be larger than ∼2 kJ mol^−1^, for the rest of the species, we do not anticipate that post-CCSD(T) corrections will substantially alter the P–F and P–Cl BDEs.

We note that the gas-phase P–F BDEs (at 298 K) of the molecules considered in this study (Table 1) differ by as much as 117.0 kJ mol^−1^, with (H_3_Si)_2_P–F being associated with the lowest BDE (439.5 kJ mol^−1^) and F_2_P–F being associated with the largest (556.5 kJ mol^−1^). Turning our attention to the P–Cl BDEs (at 298 K) of the 30 chlorophosphine-type molecules considered in this study, we note that compared with the P–F BDEs, the substituents appear to exert a smaller magnitude in terms of altering the value of the BDEs. Therefore, unlike in the case of the P–F BDEs, where a difference of 117.0 kJ mol^−1^ was noted, in the context of the P–Cl BDEs, the range of energies spans just 72.6 kJ mol^−1^. For the chlorinated species, the dicyano-substituted derivative (*i.e.*, (NC)_2_P–Cl)) is associated with the lowest P–Cl BDE (299.8 kJ mol^−1^), while substitution with two hydroxyl groups (as in (HO)_2_P–Cl) resulted in the molecule with the largest P–Cl BDE (372.4 kJ mol^−1^). Comparing the P–X (X = F and Cl) BDEs of these halophosphine-type molecules with the N–X (X = F and Cl) BDEs of halamine-type species (*i.e.*, in which the central atom sits one period above and in the same group as phosphorus) for which a previous theoretical study employing the W2 thermochemical protocol has been reported (O’Reilly et al., 2011), where comparisons can be made on the basis of available data, we note that the P–F and P–Cl bonds are evidently stronger. For example, in the case of H_2_P–F for which we compute a BDE_0_ value of 461.5 kJ mol^−1^, the corresponding N–F BDE_0_ value of H_2_N–F is 284.7 kJ mol^−1^, while the P–Cl BDE of H_2_P–Cl at 0 K (BDE_0_ = 323.4 kJ mol^−1^) is 72.1 kJ mol^−1^ higher than that of H_2_N–Cl (BDE_0_ = 251.3 kJ mol^−1^). Turning our attention now to a comparison between the five P–F and five P–Cl BDEs (*i.e.*, for H_2_PX, MeHPX, Me_2_PX, FHPX and F_2_PX) reported previously by Chan and Radom, who employed the closely-related but more economical W1 thermochemical protocol, and the BDEs obtained here using the more rigorous W2 protocol, we note that there is good agreement between the two levels. Specifically, for the P–Cl BDEs, the W1 BDEs differ by amounts ranging from 1.4 kJ mol^−1^ (in the case of MeHPCl) up to a maximum of 1.6 kJ mol^−1^ (in the case of H_2_PCl and FHPCl), with the W1 protocol underestimating the P–Cl BDEs compared with the W2 values. Concerning the P–F BDEs, the W1 values differ from the more rigorous W2 values by amounts ranging from 1.0 kJ mol^−1^ (for the BDE of FHP–F) to 1.6 kJ mol^−1^ (in the case of Me_2_P–F), but unlike in the case of the chlorinated species, we note that for the fluorophosphine-type species, the P–F BDEs obtained in conjunction with the W2 level are lower than those obtained using the W1 protocol. Our P–F BDE for PF_3_ at 0 K (552.2 kJ mol^−1^) is also in good agreement with the CCSD(T)/CBS value reported by (551.5 kJ mol^−1^) (Grant et al., 2008).

It is instructive to compare the theoretical BDEs obtained using W2 theory and experiment for the P–F and P–Cl BDEs of the trihalophosphines (*i.e.*, PF_3_ and PCl_3_). The P–F BDE of PF_3_ at 0 K obtained using the W2 thermochemical protocol (552.2 kJ mol^−1^) is in good agreement with the *D*_0_(P–F) value of 550.9 ± 1.9 kJ mol^−1^ which was obtained experimentally by way of photoionization mass spectrometry (Berkowitz et al., 1984). For PCl_3_ two experimental BDEs are available: i) 318.0 kJ mol^−1^, which was obtained by way of resonance-enhanced multiphoton ionization and time-of-flight mass spectrometry (Upadhyaya et al., 2010), and ii) 316.5 ± 14.5 kJ mol^−1^, which was obtained by way of mass spectrometry experiments based on cesium charge exchange (Mathur et al., 1976). The calculated P–Cl BDE of PCl_3_ obtained at the W2 level of theory (319.3 kJ mol^−1^ at 0 K), is in excellent agreement with the former experimental values. Given this scarcity of reliable experimental data pertaining to the strength of P–X (X = F and Cl) bonds towards homolytic dissociation, it would be desirable to conduct more experimental studies focussing on determining such quantities.

As the W2 thermochemical protocol constitutes a layered extrapolation to the relativistic all-electron CCSD(T)/CBS limit, it is insightful to consider the magnitude of the various corrections involved in the layered extrapolation. Beginning with the ΔCCSD corrections, we note that the magnitude of these corrections is significantly larger in the case of the more polar P–F vs. P–Cl BDEs, with the smallest ΔCCSD correction in the case of the P–F BDEs amounting to 123.0 kJ mol^−1^ (in the case of molecule **F7**) and the largest ΔCCSD correction in the context of the P–Cl BDEs being noted in the case of (O_2_N)PHCl (ΔCCSD = 94.8 kJ mol^−1^). Concerning the Δ(T) corrections, we note that for the P–F BDEs these range from 10.6 kJ mol^−1^ (in the case of both (NC)_2_PF and (CN)(NH_2_)PHF) to 15.0 kJ mol^−1^ in the case of (H_2_B)_2_PF, while for the P–Cl BDEs, the smallest Δ(T) correction (11.9 kJ mol^−1^) was noted in the case of the (H_2_C=CH)PHCl, while (H_2_B)_2_PCl was associated with the largest Δ(T) correction (15.8 kJ mol^−1^).

Concerning the core-valence (ΔCV) corrections, we note that for both the P–F and P–Cl BDEs, these adopt positive values. In the case of the P–Cl BDEs these differ by as much as 0.7 kJ mol^−1^, with the smallest values (0.2 kJ mol^−1^) being noted in the case of molecules **Cl12** and **Cl29**, while the largest ΔCV correction (0.9 kJ mol^−1^) arising in the case of the dissociation of (H_2_B)_2_PCl. In the context of the P–F species, the ΔCV corrections span a range of 0.8 kJ mol^−1^, with the largest correction (0.9 kJ mol^−1^) being noted in the case of (H_2_B)_2_PF. Turning our attention now to the magnitude of the scalar relativistic corrections (ΔRel) to the P–F and P–Cl BDEs, we note that for both bond types, these corrections adopt negative values and, in the case of the P–F bonds all adopt absolute values that are greater than those of the ΔCV corrections. This also holds true in the case of the P–Cl dissociations, except for (H_2_P)_2_PCl, where the ΔCV term (+0.6 kJ mol^−1^) is exactly cancelled by the ΔRel term (−0.6 kJ mol^−1^). For the dissociation of the P–F bonds, the most negative relativistic correction was noted in the case of the dissociation of (H_2_B)_2_PF (ΔRel = −2.0 kJ mol^−1^), while the least negative correction was noted in the case of the dissociation of (H_2_P)_2_PF (ΔRel = −1.0 kJ mol^−1^). Concerning the P–Cl bond dissociations, the least negative ΔRel correction was noted in the case of (HS)_2_PCl (−0.7 kJ mol^−1^), while the most negative relativistic correction was attributed to (H_2_B)_2_PCl (−1.8 kJ mol^−1^). Turning out attention to the corrections for zero-point-vibrational energy (ΔZPVE), which were obtained by way of harmonic vibrational frequency calculations at the B3LYP/A′VTZ level, we note that for the P–Cl dissociations, these corrections differ by as much as 6.1 kJ mol^−1^, with the P–Cl BDE of (HS)_2_PCl giving rise to a ΔZPVE term of the smallest magnitude (−4.8 kJ mol^−1^) and the ΔZPVE term associated with the bond dissociation of H_2_PCl (−12.9 kJ mol^−1^) being of greatest magnitude. For the P–F bond dissociations, the ΔZPVE correction of smallest magnitude was noted in the case of the dissociation of (H_3_Si)_2_PF (−7.5 kJ mol^−1^) while the largest was noted in the case of the dissociation of H_2_PF (−15.5 kJ mol^−1^). Finally, we note that with regards to the vibrational contributions to enthalpy (Δ*H*_vib_) to the P–Cl BDEs at 298 K, these corrections (which all adopt positive values) range from 2.2 kJ mol^−1^ in the case of (HS)_2_PCl to 5.2 kJ mol^−1^ in the case of H_2_PCl. On the other hand, for the P–F BDEs, the dissociation of (H_3_Si)_2_PF is associated with the smallest Δ*H*_vib_ correction (3.2 kJ mol^−1^) and H_2_PF was associated with the largest (Δ*H*_vib_ = 5.7 kJ mol^−1^).

### Effect of substituents in governing P–F and P–Cl BDEs

In this section, we examine the effect that substituents play in governing the magnitude of the P–F and P–Cl BDEs. To be able to examine the role that substituents play in altering the BDEs, it is necessary to consider the effect of substituents in both the parent halophosphine (*i.e.*, R^1^R^2^P–X) as well as the effect of substituents in the product phosphorous-centered radical (*i.e.*, R^1^R^2^P•). To assist with an analysis of such effects, we have additionally reported two quantities (both at 298 K), namely, the molecule stabilization enthalpies for both the fluorinated (MSE_PF_, Eq. 3) and chlorinated precursor molecules (MSE_PCl_, Eq. 4), which attempt to consider the effect of substituents (*i.e.*, whether they be stabilizing or destabilizing) in the context of the parent halophosphine-type molecule, while we also report so-called radical stabilization enthalpies (RSEs, Eq. 5), which consider the extent of any relative stabilizing/destabilizing effects in the product phosphorous-centered radicals.

$$R^{1}R^{2}P–F+H_{2}P–H\;\to \;R^{1}R^{2}P–H+H_{2}P–F$$ (3)

$$R^{1}R^{2}P–\text{Cl}+H_{2}P–H\;\to \;R^{1}R^{2}P–H+H_{2}P–\text{Cl}$$ (4)

$$R^{1}R^{2}P–H+H_{2}P•\;\to \;R^{1}R^{2}P•+H_{2}P–H$$ (5)

Concerning the MSEs, a positive value would be suggestive of the existence of a relative stabilizing effect in the halophosphine-precursor, while a negative value would tend to indicate that the substituents exert a relative destabilizing effect. Turning our attention to the product phosphorous-centered radicals, a negative RSE value would tend to indicate that the substituents exert a relative stabilizing effect in the product phosphorous-centered radical, while a positive value would be suggestive of the fact that the substituents exert a relative destabilizing effect on the product radical. With the MSEs and RSEs defined in this way, a thermodynamic cycle arises in which the P–X (X = F and Cl) BDE of a given halophosphine-type molecule (at 298 K) may be computed by adding the appropriate MSE and RSE for a given system to the P–X BDE of the prototypical molecule H_2_P–X (BDE_298_ = 467.2 kJ mol^−1^ for H_2_P–F and 328.6 kJ mol^−1^ for H_2_P–Cl). The MSE_PF_, MSE_PCl_ and RSE values for the species considered in this study are provided in Table 3, as well as the equilibrium P–F and P–Cl bond lengths (*r*_P–F_ and *r*_P–Cl_ in Å).

**TABLE 3:** Molecule Stabilization Enthalpies of the fluorophosphine-type (MSE_PF_) and chlorophosphine-type (MSE_PCl_) reactants, Radical Stabilization Enthalpies of the phosphorus-centered radicals (RSE) (at 298 K in kJ mol^−1^), as well as equilibrium bond distances of the P–F (*r*_P–F_) and P–Cl (*r*_P–Cl_) bonds in the reactant halophosphines (in Å).

R^1^	R^2^	MSE_PF_	MSE_PCl_	RSE	*r*_P–F_	*r*_P–Cl_
SiH_3_	SiH_3_	−22.9	−1.1	−4.8	1.643	2.115
SiH_3_	H	−12.4	−1.2	−2.4	1.631	2.102
BH_2_	H	−5.1	−3.4	10.1	1.616	2.079
H	H	0.0	0.0	0.0	1.619	2.088
BH_2_	BH_2_	1.6	0.7	20.1	1.599	2.048
CN	H	3.9	−2.7	−13.2	1.603	2.073
CN	CN	8.3	−3.5	−25.3	1.591	2.061
CHO	H	9.1	9.4	−4.4	1.617	2.081
CH_3_	SiH_3_	10.7	18.4	−7.0	1.637	2.109
PH_2_	H	14.3	12.9	−15.7	1.624	2.098
HC≡C	H	14.8	7.6	−22.8	1.615	2.093
PH_2_	PH_2_	21.0	21.8	−28.1	1.630	2.106
H_2_C=CH	H	22.1	18.1	−23.8	1.626	2.103
CH_3_	H	25.4	21.1	−5.3	1.625	2.097
NO_2_	H	26.1	13.8	−0.7	1.590	2.047
SH	H	34.6	21.6	−29.4	1.619	2.103
Cl	H	36.1	20.7	−20.8	1.604	2.077
SH	SH	43.1	20.6	−33.1	1.616	2.115
CH_3_	CH_3_	47.0	39.0	−9.2	1.631	2.106
CH_3_	Cl	55.9	36.1	−24.2	1.609	2.087
CN	NH_2_	57.5	31.1	−33.2	1.616	2.115
F	H	61.8	36.1	−15.1	1.598	2.072
Cl	Cl	64.4	33.5	−40.3	1.590	2.072
OH	H	65.0	43.5	−22.2	1.613	2.104
NH_2_	H	65.3	46.9	−27.0	1.635	2.137
NH_2_	Cl	68.5	35.8	−19.0	1.602	2.122
CH_3_	F	81.5	51.5	−19.6	1.605	2.086
NH_2_	NH_2_	82.2	51.5	−20.5	1.629	2.150
OH	OH	107.6	65.1	−21.4	1.614	2.133
F	F	118.1	65.3	−28.8	1.581	2.068

Before proceeding to discuss trends concerning the effect of the selected substituent(s) in governing the strength of the P–F and P–Cl BDEs toward homolytic dissociation, we begin initially by making several general points concerning the extent to which the substituents govern the molecule stabilization enthalpies (MSE_PF_ and MSE_PCl_) as well as the radical stabilization enthalpies (RSEs). First, concerning the MSE_PF_ and MSE_PCl_ values, we note that for the most part, these adopt positive values, indicating that there is a tendency for the substituents to stabilize the parent halophosphine molecules. In the context of the fluorophosphine-type species, the largest MSE_PF_ values were noted in the case of F_2_P–F and (HO)_2_P–F (MSE_PF_ = 118.1 and 107.6 kJ mol^−1^, respectively). The only P–F-containing molecules that were associated with negative MSE_PF_ values were (H_2_B)HP–F (−5.1 kJ mol^−1^), (H_3_Si)HP–F (−12.4 kJ mol^−1^) and (H_3_Si)_2_P–F (−22.9 kJ mol^−1^). Turning our attention now to the MSE_PCl_ values, we note that, as was noted in the case of the P–F-containing species, substitution with either two fluorine (as in F_2_P–Cl) or two hydroxy (as in (HO)_2_P–Cl) substituents resulted in the greatest degree of stabilization, with these two molecules being associated with MSE_PCl_ values of 65.3 and 65.1 kJ mol^−1^, respectively). Concerning the effect of substituents in the product radicals, we note that the RSE values are nearly all negative (ranging from −0.7 kJ mol^−1^ in the case of (O_2_N)HP• to −40.3 kJ mol^−1^ in the case of Cl_2_P•), with the exception of the boron-substituted radicals, namely, (H_2_B)HP• and (H_2_B)_2_P•, for which we computed RSE values of +10.1 and +20.1 kJ mol^−1^, respectively.

Turning our attention now to the effect of substituents in governing the P–F BDEs, we will initially consider the variation in BDEs upon attaching a single substituent with an atom belonging to Period 2 (and the valence of the substituent atom being fulfilled through the attachment of hydrogen atom(s), where applicable). For this set of species, we attain the following P–F BDEs at 298 K (H_2_B)HP–F (472.2) < (H_3_C)HP–F (487.3) < (H_2_N)HP–F (505.6) < (HO)HP–F (510.1) < FHP–F (513.9 kJ mol^−1^). Thus, we see that the P–F BDEs of the monosubstituted species increase monotonically as the electronegativity of the atom directly attached to the phosphorous atom increases. We note that the same trend also holds when looking at the variation in BDEs as one considers the P–F BDEs of those species containing an atom from Period Three directly attached to the central phosphorus atom, thus for these species, we obtain P–F BDEs at 298 K of (H_3_Si)HP–F (452.4) < (H_2_P)HP–F (465.8) < (HS)HP–F (472.5) < ClHP–F (482.5 kJ mol^−1^). Concerning the P–F BDEs of the disubstituted species (*i.e.*, R_2_P–F), for those molecules that contain substituents belonging to the second period, we obtain the following values (in kJ mol^−1^ and at 298 K) (H_2_B)_2_P–F (488.9) < (H_3_C)_2_P–F (505.0) < (H_2_N)_2_P–F (528.9) < (HO)_2_P–F (553.5) < F_2_P–F (556.5), while for those containing substituents belonging to the third period, we obtain the following P–F BDEs (in kJ mol^−1^ and at 298 K) (H_3_Si)_2_P–F (439.5) < (H_2_P)_2_P–F (460.1) < (HS)_2_P–F (477.3) < Cl_2_P–F (491.3). We note that the relative effect of the substituents in governing the P–F BDEs in the disubstituted molecules are qualitatively consistent with the trends observed in the analogous monosubstituted species.

We now turn our attention to rationalizing the variation in the P–F BDEs of molecules containing second-period elements bonded directly to the central phosphorus atom. The increased BDE of (H_2_B)HP–F (472.2 kJ mol^−1^), compared with that of H_2_P–F (467.2 kJ mol^−1^), can be accounted for on the basis that this substituent exerts a relative destabilizing effect in the product radical (H_2_B)HP• (RSE = +10.1 kJ mol^−1^), that is of slightly greater magnitude than the extent to which it exerts a relative destabilizing effect in the fluorinated-precursor molecule (H_2_B)HP–F (MSE_PF_ = −5.1 kJ mol^−1^). In replacing the -BH_2_ substituent with a -CH_3_ group, we see a further increase in the P–F BDE (by 15.1 kJ mol^−1^ compared with that of (H_2_B)HP–F) which can be reconciled on the basis that the extent of stabilization afforded by the methyl substituent in (H_3_C)HPF (MSE_PF_ = +25.4 kJ mol^−1^) is of greater magnitude than the degree of stabilization that it affords in (H_3_C)HP• (RSE = −5.3 kJ mol^−1^). In comparing the effect of the -NH_2_, -OH and -F substituents on the P–F BDEs, we note that while the MSE_PF_ values vary by just 3.5 kJ mol^−1^, the variation in the magnitude of stabilizing effects in the product radicals (as measured by way of the RSEs) show a much greater variation (by up to 11.9 kJ mol^−1^). Consequently, the variation in the P–F BDEs of these three molecules is governed predominantly by the relative degree to which the substituents exert stabilizing effects in the product phosphorus-centered radicals, with the RSE of FHP• being the least negative (−15.1 kJ mol^−1^), followed by that of (HO)HP• (−22.2 kJ mol^−1^), and (H_2_N)HP• (−27.0 kJ mol^−1^).

Regarding the attachment of the other substituents in which carbon bonds to the central phosphorus atom, we note that in comparing the effect of modification of the hybridization of the carbon atom in the hydrocarbon-based substituents, attachment of an alkyl group (as in (H_3_C)HP–F) gives rise to a larger P–F BDE (487.3 kJ mol^−1^) than those species containing either an alkenyl substituent (as in (H_2_C=CH)HP–F, for which we compute a P–F BDE of 465.6 kJ mol^−1^) or an alkynyl substituent (as in (HC≡C)HP–F, for which we compute a P–F BDE of 459.2 kJ mol^−1^). Given that the MSE_PF_ values of both (H_3_C)HP–F and (H_2_C=CH)HP–F do not vary significantly (adopting values of 25.4 and 22.1 kJ mol^−1^, respectively), the significantly larger BDE of (H_3_C)HP–F arises, therefore, because of the considerably smaller extent to which the methyl group is able to stabilize the product radical (*i.e* (H_3_C)HP•), compared with the alkenyl substituent in (H_2_C=CH)HP•, which allows for delocalization of the unpaired electron (in this regard, we note that the terminal carbon atom of the alkenyl substituent is associated with a Mulliken spin density of 0.355, based on computations performed at the B3LYP/A′VTZ level of theory). This is reflected in the RSE values of these two radicals, with the RSE of (H_2_C=CH)HP• (−23.8 kJ mol^−1^) being significant more negative than that of (H_3_C)HP• (−5.3 kJ mol^−1^). In comparing the P–F BDEs of the alkenyl- and alkynyl-substituted species, we note that whereas the product radicals are associated with RSEs that differ by just 1.0 kJ mol^−1^, the smaller P–F BDE of (HC≡C)HP–F (459.2 kJ mol^−1^) compared with that of (H_2_C=CH)HP–F (465.6 kJ mol^−1^) arises consequently because of the greater degree to which the alkenyl substituent stabilizes the fluorinated precursor molecule compared with the alkynyl-substituted system (with MSE_PF_ values of 22.1 and 14.8 kJ mol^−1^, respectively). We note that attachment of a single cyano substituent (as in (NC)HP–F) results in a P–F BDE (458.0 kJ mol^−1^) that is comparable to that of (HC≡C)HP–F (459.2 kJ mol^−1^), while attachment of a single electron-withdrawing formyl substituent (as in (HCO)HP–F) results in a P–F BDE of 471.9 kJ mol^−1^.

We now turn our attention to the effect of substituents in governing the magnitude of the gas-phase homolytic P–Cl BDEs (at 298 K) of the chlorophosphine-type species. Prior to commenting on trends that exist concerning the P–Cl BDEs, we initially note that the significantly larger range of P–F vs. P–Cl BDEs at 298 K (117.0 vs. 72.6 kJ mol^−1^, respectively), arises because of especially large stabilizing effects in F_2_P–F, (HO)_2_P–F, and to a lesser extent (H_2_N)_2_P–F (MSE_PF_ = 118.1, 107.6 and 82.2 kJ mol^−1^, respectively), which were of noticeably greater magnitude than the corresponding effects in the chlorinated derivatives, for which the MSE_PCl_ values of the corresponding chlorinated derivatives were found to be 65.3, 65.1, and 51.5 kJ mol^−1^, respectively. The lowest P–Cl BDE was noted in the case of the (CN)_2_P–Cl (299.8 kJ mol^−1^), and the relatively low strength of this bond toward dissociation can be accounted for on the basis that: (i) the two cyano substituents exert a slight destabilizing effect in the chlorinated precursor (MSE_PCl_ = −3.5 kJ mol^−1^), while (ii) the two cyano substituents exert a significant stabilizing effect in (NC)_2_P• (RSE = −25.3 kJ mol^−1^). The largest P–Cl BDE was noted in the case of (OH)_2_P–Cl (372.4 kJ mol^–1^ at 298 K).

Turning our attention now to the P–Cl BDEs of the monosubstituted chloroborane-type species (*i.e.*, R^1^HP–Cl) containing substituent atoms that belong to Period Two (and having their valence reached by adding hydrogen atom(s) as necessary), we obtain the following BDEs at 298 K (H_2_B)HP–Cl (335.4) < (H_3_C)HP–Cl (344.4) < (H_2_N)HP–Cl (348.6) < FHP–Cl (349.7) < (HO)HP–Cl (350.0 kJ mol^−1^). For the analogous disubstituted species (*i.e.*, R_2_P–Cl), we obtain the same general trend (in terms of the effect of substituents in governing the relative ordering of the P–Cl BDEs) as was observed in the case of the monosubstituted systems. Thus, for the disubstituted species, we note that the P–Cl BDEs (in kJ mol^−1^ and at 298 K) increase in the order (H_2_B)_2_P–Cl (349.4) < (H_3_C)_2_P–Cl (358.4) < (H_2_N)_2_P–Cl (359.6) < F_2_P–Cl (365.1) < (HO)_2_P–Cl (372.4).

Concerning the relative ordering of P–Cl BDEs in the case of molecules that have been monosubstituted with groups bearing atoms from Period Three (*i.e.*, -SiH_3_, -PH_2_, -SH, and -Cl), we note that unlike in the case of the P–F BDEs, where a monotonic variation of the P–F BDEs with increasing electronegativity of the substituent atom directly attached to the phosphrous atom was noted, a strictly monotonic trend is not observed in the case of the dissociation of the P–Cl bonds of the corresponding chlorophosphine-type molecules. Indeed, for this set of four molecules, we obtain P–Cl BDEs (at 298 K) of (H_3_Si)HP–Cl (325.0) → (H_2_P)HP–Cl (325.8) → (HS)HP–Cl (320.8) → ClHP–Cl (328.5 kJ mol^−1^). For the corresponding disubstituted species, we obtain the following P–Cl BDEs (at 298 K) (HS)_2_P–Cl (316.2) < Cl_2_P–Cl (321.8) < (H_2_P)_2_P–Cl (322.4) < (H_3_Si)_2_P–Cl (322.7 kJ mol^−1^).

### Performance of DFT methods for the computation of P–F and P–Cl BDEs

Although the W2 thermochemical protocol offers a robust approach to obtaining quantitively accurate thermochemical data, it does come at a significant computational cost. As such, its use is necessarily limited to the study of relatively small molecules. On the other hand, a plethora of lower-cost DFT methods are available, and which may be used to study the thermochemistry of much larger molecules. Having said that, given the approximate nature of the DFT and the plethora of available DFT methods, it is not *a priori* clear which functional will perform well for the BDEs at hand. Here, we investigate the performance of a wide range of DFT methods for their ability to compute gas-phase P–X BDEs. As reference values, we have used the complete set of all-electron, non-relativistic P–X BDEs (*i.e.*, those that are arrived at by adding the ΔSCF, ΔCCSD, Δ(T) and ΔCV components). We have examined the performance of a diverse selection of functionals belonging to each rung of Jacob’s Ladder, including seven GGAs, eight MGGAs, fifteen HGGAs, eleven HMGGAs, and ten double-hybrid DFT methods. Where available, we have augmented these functionals with Becke-Johnson (D3BJ) dispersion corrections, to investigate the effect that such corrections have in altering the performance of the underlying non-corrected functionals. We have performed these calculations in conjunction with the A′VQZ basis set, which is expected to give BDEs close to the basis-set limit for the conventional functionals and is also sufficiently large for the DHDFT calculations (Karton and Martin, 2011). For each functional we have reported a mean absolute deviation (MAD), mean deviation (MD), largest deviation (LD), as well as the number of outliers (NO, which are defined as the number of BDEs which deviate from the W2 reference values by ≥10 kJ mol^–1^). We present these results in Table 4.

**TABLE 4:** Performance of a Range of DFT Methods for the Computation of Gas-Phase Homolytic P–F and P–Cl Bond Dissociation Energies (BDEs) in Conjunction with the A′VQZ basis set (in kJ mol^−1^).

		P–F BDEs				P–Cl BDEs				
Class	Functional	MAD	MD	LD	NO	MAD		MD	LD	NO
GGA	revPBE	25.8	−25.8	37.9 (**F30**)	29	23.3		−23.3	29.0 (**Cl1**)	29
	revPBE-D3BJ	20.4	−20.4	33.1 (**F30**)	28	8.8		−8.8	13.6 (**Cl29**)	11
	B97-D	16.1	−16.1	29.0 (**F30**)	25	31.4		−31.4	39.3 (**Cl1**)	29
	HCTH407	13.3	−13.3	22.3 (**F30**)	22	26.4		−26.4	32.9 (**Cl1**)	29
	BLYP	12.3	−12.2	26.9 (**F30**)	18	31.6		−31.6	38.9 (**Cl1**)	29
	BPW91	11.9	−11.9	23.4 (**F30**)	18	15.7		−15.7	21.9 (**Cl1**)	29
	BLYP-D3BJ	8.4	−7.7	23.0 (**F30**)	10	17.4		−17.4	23.5 (**Cl1**)	29
	PBE-D3BJ	6.2	4.8	15.1 (**F1**)	5	4.6		4.4	8.5 (**Cl14**)	0
	BP86-D3BJ	5.2	2.3	13.5 (**F1**)	3	3.5		2.5	7.6 (**Cl8**)	0
	PBE	5.1	2.6	12.7 (**F1**)	4	3.0		−1.8	8.1 (**Cl1**)	0
	BP86	5.0	−1.0	12.1 (**F30**)	3	9.1		−9.1	15.9 (**Cl1**)	13
MGGA	M11-L	25.1	−25.1	29.7 (**F17**)	29	3.2		−1.9	9.8 (**Cl23**)	0
	*r* ^2^SCAN	14.9	−14.9	21.6 (**F2**)	27	9.7		−9.7	16.9 (**Cl1**)	12
	TPSS	14.7	−14.7	26.8 (**F30**)	23	15.9		−15.9	22.0 (**Cl1**)	29
	TPSS-D3BJ	12.0	−12.0	24.6 (**F30**)	18	7.6		−7.6	12.9 (**Cl1**)	8
	B97M-V	10.9	−10.9	18.8 (**F2**)	12	2.9		0.2	8.2 (**Cl1**)	0
	VSXC	9.3	−9.3	19.5 (**F19**)	10	18.2		−18.2	29.7 (**Cl1**)	28
	*τ*-HCTH	8.2	−8.2	17.2 (**F30**)	11	24.1		−24.1	31.7 (**Cl1**)	29
	MN12-L	7.6	−7.6	15.8 (**F2**)	8	4.2		0.6	10.3 (**Cl29**)	3
	MN15-L	3.8	−3.1	9.5 (**F2**)	0	5.6		5.5	12.1 (**Cl23**)	1
HGGA	BH&HLYP	49.0	−49.0	54.8 (**F30**)	29	42.9		−42.9	47.6 (**Cl1**)	29
	SOGGA11-X	23.0	−23.0	27.7 (**F30**)	29	11.0		−11.0	15.1 (**Cl1**)	16
	B3PW91	22.4	−22.4	30.2 (**F30**)	29	18.9		−18.9	23.8 (**Cl1**)	29
	APF	22.0	−22.0	28.8 (**F30**)	29	14.7		−14.7	19.4 (**Cl1**)	29
	PBE0	21.7	−21.7	27.9 (**F30**)	29	11.8		−11.8	16.3 (**Cl1**)	22
	PBE0-D3BJ	19.9	−19.9	26.4 (**F30**)	29	6.0		−6.0	9.9 (**Cl1**)	0
	B3LYP	19.9	−19.9	30.2 (**F30**)	29	29.8		−29.8	35.6 (**Cl1**)	29
	B3PW91-D3BJ	18.7	−18.7	27.0 (**F30**)	29	7.0		−7.0	11.3 (**Cl29**)	4
	APF-D	18.5	−18.5	24.8 (**F30**)	29	10.6		−10.6	15.2 (**Cl1**)	16
	X3LYP	18.4	−18.4	28.3 (**F30**)	28	27.8		−27.8	33.5 (**Cl1**)	29
	B3LYP-D3BJ	16.2	−16.2	27.1 (**F30**)	27	18.0		−18.0	23.0 (**Cl29**)	29
	ωB97X-D	14.2	−14.2	22.4 (**F30**)	27	11.2		−11.2	15.3 (**Cl21**)	20
	B971	12.0	−12.0	20.2 (**F30**)	20	8.6		−8.6	14.9 (**Cl1**)	14
	ωB97X	10.2	−10.2	18.5 (**F30**)	15	12.8		−12.8	16.2 (**Cl29**)	27
	ωB97	7.6	−7.6	17.3 (**F30**)	6	15.5		−15.5	20.2 (**Cl29**)	29
	CAM-B3LYP	7.3	−7.3	16.8 (**F30**)	8	27.3		−27.3	31.4 (**Cl21**)	29
	CAM-B3LYP-D3BJ	5.8	−5.7	15.5 (**F30**)	3	21.5		−21.5	26.4 (**Cl29**)	29
	ωB97X-V	4.1	−4.0	13.0 (**F30**)	2	5.6		−5.6	10.1 (**Cl30**)	1
	N12-SX	3.2	−0.6	8.0 (**F2**)	0	4.7		4.4	11.8 (**Cl14**)	2
HMGGA	TPSSh	23.2	−23.2	33.4 (**F30**)	29	18.8		−18.8	24.1 (**Cl1**)	29
	PW6B95	9.2	−9.2	16.4 (**F30**)	13	8.3		−8.3	14.0 (**Cl12**)	11
	MN15	8.6	8.6	18.8 (**F17**)	9	3.5		−3.1	10.1 (**Cl1**)	1
	PW6B95-D3BJ	8.1	−8.1	15.6 (**F30**)	8	4.4		−4.3	9.7 (**Cl12**)	0
	*τ*-HCTHh	8.0	−8.0	16.6 (**F30**)	10	11.9		−11.9	19.0 (**Cl1**)	18
	BMK	5.8	−5.8	10.7 (**F30**)	3	2.3		−0.6	6.3 (**Cl14**)	0
	M06-2X	4.6	−4.6	7.5 (**F2**)	0	4.8		−4.8	8.1 (**Cl1**)	0
	M08-HX	3.8	−3.7	8.8 (**F30**)	0	6.8		−6.8	10.5 (**Cl29**)	1
	M06	3.7	−3.4	10.3 (**F19**)	1	3.8		3.1	7.8 (**Cl14**)	0
	BMK-D3BJ	3.6	−3.5	8.7 (**F30**)	0	10.0		10.0	15.0 (**Cl8**)	14
	M05-2X	3.0	2.6	7.2 (**F9**)	0	6.0		−6.0	10.7 (**Cl21**)	1
	M11	3.0	−1.9	8.5 (**F30**)	0	12.9		−12.9	18.7 (**Cl29**)	24
	ωB97M-V	2.2	1.4	5.5 (**F6**)	0	2.4		2.3	4.8 (**Cl14**)	0
DHDFT	PBE0-DH	23.5	−23.5	26.7 (**F30**)	29	9.7		−9.7	11.6 (**Cl1**)	15
	PBE-QIDH	12.1	−12.1	14.2 (**F30**)	27	2.3		−2.1	4.0 (**Cl29**)	0
	mPW2PLYP	10.1	−10.1	16.7 (**F30**)	14	16.1		−16.1	19.0 (**Cl21**)	29
	B2PLYP	8.8	−8.8	16.1 (**F30**)	10	16.3		−16.3	19.4 (**Cl21**)	29
	B2GP-PLYP	7.4	−7.4	13.1 (**F30**)	4	12.6		−12.6	15.8 (**Cl29**)	25
	B2PLYP-D3BJ	7.2	−7.2	14.6 (**F30**)	6	10.3		−10.3	14.7 (**Cl29**)	13
	B2K-PLYP	6.1	−6.1	11.0 (**F30**)	1	10.3		−10.3	13.5 (**Cl29**)	21
	DSD-BLYP	5.3	5.3	13.3 (**F2**)	1	2.4		−1.0	7.8 (**Cl1**)	0
	DSD-PBEP86	5.2	−5.0	9.9 (**F30**)	0	2.4		−0.9	7.8 (**Cl1**)	0
	PWPB95	5.0	−5.0	11.1 (**F30**)	2	4.8		−4.8	7.8 (**Cl27**)	0
	DSD-PBEB95	2.1	1.7	6.7 (**F2**)	0	2.2		2.1	7.9 (**Cl1**)	0

Before embarking on a discussion of the performance of the functionals within each rung of Jacob’s Ladder, we first wish to highlight a few general findings. First, of all the functionals investigated, we note that only 10 attain MADs below the threshold of chemical accuracy (i.e., MADs ≤4.2 kJ mol^−1^) (Karton, 2022) for the computation of the P–F BDEs, while for the P–Cl BDEs 13 functionals fall within this threshold. Second, we find that the double-hybrid functional DSD-PBEB95 offers the best overall performance for the computation of P–F and P–Cl BDEs, with MADs for the computation of the P–F and P–Cl BDEs amounting to just 2.1 and 2.2 kJ mol^−1^, respectively (and with LDs of 6.7 and 7.9 kJ mol^−1^, respectively). In contrast, the worst performing method was shown to be BH&HLYP, with a MAD of 49.0 kJ mol^−1^ for the computation of the P–F BDEs and a MAD of 42.9 kJ mol^−1^ in the case of the P–Cl BDEs. The poor performance of BH&HLYP in this assessment study is consistent with its performance in the computation of N–F, N–Cl, N–Br and B–Cl BDEs, where it attained MADs of 62.2, 53.0, 55.3 and 36.5 kJ mol^−1^, respectively (O'Reilly et al., 2012; Akhmetova et al., 2016; O’Reilly and Karton, 2016; Lu and O’Reilly, 2022). Third, adding the Becke-Johnson D3 dispersion correction, for the most part, tends to improve the performance of the functionals by amounts ranging from 1.1 (PW6B95) to 5.4 kJ mol^−1^ (revPBE) in the case of the P–F BDEs, and by 4.0 (PW6B95) to 14.5 kJ mol^−1^ (revPBE) for the P–Cl BDEs. Nevertheless, in some cases the inclusion of the dispersion correction increases the MADs compared with the unmodified parent functionals. In the case of the P–F BDEs, addition of the D3BJ correction to BP86 and PBE result in MAD increases by 0.2 and 1.1 kJ mol^−1^, respectively. In the case of the P–Cl BDEs, performance deterioration is noted in the case of PBE and BMK, where the MADs are increased by 1.6 and 7.7 kJ mol^−1^, respectively. Fourth, in the case of the P–F BDEs, the largest deviation in the case of approximately three-quarters of the functionals was observed in the case of molecule **F30** (*i.e.*, PF_3_), while for the P–Cl BDEs, approximately half of the functionals found the BDE of molecule **Cl1** (*i.e* (NC)_2_PCl) to be the most challenging.

Considering the performance of the GGA functionals, we note that for the P–F BDEs, BP86 offered the best performance with an MAD of 5.0 kJ mol^−1^ and an LD of 12.1 kJ mol^−1^ (in the case of molecule **F30**), followed very closely by PBE, which afforded an MAD and LD that were just 0.1 and 0.6 kJ mol^−1^ higher than that obtained with BP86, respectively. In addition, while BP86 had a slight tendency to underestimate the P–F BDEs (with an MD of −1.0 kJ mol^−1^), PBE tended to overestimate them (MD = +2.6 kJ mol^−1^). In the context of the P–Cl BDEs, we found that PBE offered the best performance, with a MAD of 3.0 kJ mol^−1^ and an LD of 8.1 kJ mol^−1^ (in the case of molecule **Cl1**). The performance of PBE for the computation of the P–Cl BDEs was slightly better than its performance across the set of P–F BDEs (with a MAD reduction of 2.1 kJ mol^−1^). Beyond PBE, the next best performing method for the computation of P–Cl BDEs was found to be BP86-D3BJ (MAD = 3.5 kJ mol^−1^). For the computation of P–Cl BDEs, the inclusion of the D3BJ correction in the case of BP86 results in a performance improvement of 5.6 kJ mol^−1^ compared with the uncorrected functional, although for the computation of P–F BDEs, the inclusion of the D3BJ correction to the BP86 functional resulted in a deterioration in performance (with an MAD that was 0.2 kJ mol^−1^ higher than that obtained with the parent BP86 functional). The worst performing functional for the computation of the P–F BDEs was found to be revPBE (MAD = 25.8 kJ mol^−1^ and LD = 37.9 kJ mol^−1^) while for the P–Cl BDEs, the worst performing methods were found to be BLYP (MAD = 31.6 kJ mol^−1^) and B97-D (MAD = 31.4 kJ mol^−1^).

We now turn our attention to the performance of the meta-GGA functionals (MGGAs), which include the kinetic energy density. We have examined the performance of nine such methods. In examining the performance of the selected MGGAs in the context of the computation of P–F BDEs, we note that the functionals offer MADs that range from 3.8 kJ mol^−1^ (in the case of MN15-L) up to 25.1 kJ mol^−1^ (in the case of M11-L). The MN12-L functional was the second-best performing method with an MAD (7.6 kJ mol^−1^) that was 3.8 kJ mol^−1^ higher than that of MN15-L, and an LD that was 6.3 kJ mol^−1^ higher than that of MN15-L, although both functionals appeared to find the computation of the P–F BDE of molecule **F2** to be the most challenging. It is of interest to note that although M11-L was clearly the worst performing MGGA functional in the context of the computation of P–F BDEs (the second worst performing MGGA was *r* ^2^SCAN, with an MAD that was lower than that of M11-L by 10.2 kJ mol^−1^) it was the second-best performer when it came to the computation of the P–Cl BDEs (with an MAD of 3.2 kJ mol^−1^ and an LD of 9.8 kJ mol^−1^). Turning our attention now then to the P–Cl BDEs, the best performing method was shown to be B97M-V (MAD = 2.9 and LD = 8.2 kJ mol^−1^). The worst performing functionals for the computation of P–Cl BDEs were shown to be VSXC and *τ*-HTCH with MADs of 18.2 and 24.1 kJ mol^−1^, respectively. Concerning the effect of D3BJ corrections in the context of MGGA functionals, this was only examined in the context of the TPSS functional, where we note that inclusion of this correction served to lower the MADs by 2.7 kJ mol^−1^ (in the case of the P–F BDEs) and 8.3 kJ mol^−1^ (in the case of the P–Cl BDEs) compared with the MADs of the unmodified parent TPSS functional.

Moving up one more rung of Jacob’s Ladder to the HGGA functionals, we note that BH&HLYP offers by far the worst performance across both the P–F and P–Cl BDEs, with MADs of 49.0 and 42.9 kJ mol^−1^, respectively. Of the HGGA functionals considered, the best-performing methods for the computation of both the P–F and P–Cl BDEs were shown to be N12-SX and ωB97X-V. Beginning with their performance for the calculation of P–F BDEs, we obtained MADs of 3.2 and 4.1 kJ mol^−1^, respectively. The same performance ordering was noted in the case of the P–Cl BDEs (MADs = 4.7 and 5.6 kJ mol^−1^ for N12-SX and ωB97M-V, respectively), although the functionals performed slightly worse in the context of the P–Cl vs. P–F BDEs (with MADs that were raised by 1.5 kJ mol^−1^ in both cases). We note that the CAM-B3LYP functional performed substantially better for the computation of the P–F BDEs (MAD = 7.3 kJ mol^−1^) compared with its performance across the dataset of P–Cl BDEs (MAD = 27.3 kJ mol^−1^). This difference in MAD, amounting to 20.0 kJ mol^−1^, was the largest difference seen for any of the HGGAs regarding their comparative performance in the context of both bond types. Concerning the inclusion of a D3BJ dispersion correction, we examined this in the context of four HGGA functionals (PBE0, B3PW91, B3LYP and CAM-B3LYP), and note that in all cases, the inclusion of this contribution served to improve the performance of the underlying functionals (*i.e.*, to lower the MADs of the corrected vs. uncorrected functionals). For the P–F BDEs, the magnitude of these performance improvements ranged from 1.5 kJ mol^−1^ (in the case of CAM-B3LYP) to 3.7 kJ mol^−1^ (in the case of both B3LYP and B3PW91). The extent of performance improvements in the case of the P–Cl BDEs was significantly higher, with MADs that were reduced by between 5.8 kJ mol^−1^ (in the case of both PBE0 and CAM-B3LYP) and 12.0 kJ mol^−1^ (in the case of B3PW91).

Turning our attention to the HMGGA functionals, we note that the best performing of these for the computation of P–F BDEs was ωB97M-V (MAD = 2.2 and LD = 5.5 kJ mol^−1^). This functional also offered the second-best performance for the computation the P–Cl BDEs (MAD = 2.4 and LD = 4.8 kJ mol^−1^). In fact, the best-performing method when assessed against the dataset of P–Cl BDEs, namely, BMK, had an MAD (2.3 kJ mol^−1^) that was just 0.1 kJ mol^−1^ lower than that of ωB97M-V. Having said that, ωB97M-V offered a significantly lower LD (4.8 kJ mol^−1^) compared with BMK (LD = 6.3 kJ mol^−1^), with both functionals experiencing the greatest challenge in computing the P–Cl BDE of molecule **Cl14**. The worst performing HMGGA functional for the computation of both P–F and P–Cl BDEs was shown to be TPSSh, which attained an MAD of 23.2 kJ mol^−1^ when assessed against the dataset of P–F BDEs, and an MAD of 18.8 for the computation of the P–Cl BDEs. It is of interest to note that the M11 functional, which was the second-best performing functional for the computation of the P–F BDEs (MAD = 3.0 kJ mol^−1^), offered significantly worse performance for the computation of the P–Cl BDEs (MAD = 12.9 kJ mol^−1^). Concerning the inclusion of the D3BJ correction to the HMGGAs, this was examined in the context of both PW6B95 and BMK, and inclusion of such a correction for both functionals resulted in a reduction in the MADs (compared with the MADs of the parent functionals for the computation of the P–F BDEs) by 1.1 and 2.2 kJ mol^−1^, respectively. In the context of the P–Cl BDEs, the D3BJ correction served to improve the performance of PW6B95, lowering the MAD by 3.9 kJ mol^−1^ compared with the parent functional, but interestingly, when the D3BJ correction was applied to the BMK functional, a deterioration in performance was noted. Thus, the MAD of BMK-D3BJ was 7.7 kJ mol^−1^ higher than that of BMK.

Finally, we wish to examine the performance of a range of double-hybrid DFT methods for their ability to compute P–F and P–Cl BDEs. The best performing method for both bond types was shown to be DSD-PBEB95, which attained an MAD of 2.1 kJ mol^−1^ in the case of the P–F BDEs, and an MAD of 2.2 kJ mol^−1^ in the context of the P–Cl BDEs (the LDs for this functional across both bond types were determined to be 6.7 and 7.9 kJ mol^−1^, respectively). DSD-PBEB95 offered the best overall performance of all the functionals belonging to any rungs of Jacob’s Ladder. Regarding the comparative performance of the DHDFTs for both bond types, we note that for the P–F BDEs, only one functional (DSD-PBEB95) attained an MAD ≤4.2 kJ mol^−1^, whereas for the P–Cl BDEs, four DHDFT methods achieved this threshold. The worst performing DHDFT method for the computation of the P–F BDEs was PBE0-DH, with a MAD of 23.5 kJ mol^−1^, although this method performed significantly better (although still largely unacceptably) for the computation of P–Cl BDEs (MAD = 9.7 kJ mol^−1^). The worst performing method for the computation of the P–Cl BDEs was shown to be B2PLYP, with a MAD and LD of 16.3 and 19.4 kJ mol^−1^, respectively. Inclusion of the D3BJ correction to B2PLYP served to lower the MADs when assessed against both the P–F and P–Cl BDE datasets, with MAD reductions of 1.6 and 6.0 kJ mol^−1^, respectively.

## Conclusion

Both fluorophosphine and chlorophosphine-type molecules (*i.e.*, R^1^R^2^P–X, where X = F or Cl) are useful reagents in organic synthesis and as ligands in transition metal complexes. In this study, we sought to examine the effect of substituents in governing the strength of the P–F and P–Cl bonds of these types of compounds toward homolytic bond dissociation, affording a halide atom and a phosphorous-centered radical. To achieve this, we employed the high-level W2 thermochemical protocol and used it in obtaining a set of 30 gas-phase homolytic P–F and 30 gas-phase homolytic P–Cl BDEs. For the fluorophosphine-type species, we note that the P–F BDEs (at 298 K) differ by as much as 117.0 kJ mol^−1^, with the disilyl-substituted molecule (H_3_Si)_2_P–F having the lowest P–F BDE (439.5 kJ mol^−1^) and F_2_P–F having the largest (556.5 kJ mol^−1^). Concerning the P–Cl BDEs (at 298 K), we note that these varied by a smaller amount (up to 72.6 kJ mol^−1^), with the dicyano-substituted molecule (NC)_2_P–Cl having the lowest P–Cl BDE (299.8 kJ mol^−1^) and (HO)_2_P–Cl having the highest P–Cl BDE (372.4 kJ mol^−1^). We note that for both the monosubstituted fluorophosphines (*i.e.*, RHP–F) and analogous disubstituted species (*i.e.*, R_2_P–F) which contain substituent atoms belonging to either Period 2 or Period 3 (with the valence of the said atoms being fulfilled through the attachment of hydrogen atom(s)), a monotonic variation in the P–F BDEs (from small to large) exists as one moves from left to right across each period. For example, in the case of species containing a substituent atom belonging to Period 2, we attain the following variation in P–F BDEs (at 298 K, and expressed in kJ mol^−1^) (H_2_B)HP–F (472.2) < (H_3_C)HP–F (487.3) < (H_2_N)HP–F (505.6) < (HO)HP–F (510.1) < FHP–F (513.9). On the other hand, such strictly monotonic variations in the P–Cl BDEs of the corresponding mono- or disubstituted-chlorophosphine-type molecules were not observed. For both the fluorophosphine and chlorophosphine-type molecules, we note that in considering the effect of substituents that belong to the same group, substitution with Period 2 *versus* 3 elements give rise to larger P–X (X = F and Cl) BDEs. We additionally examined the effect of substituents in governing the magnitude of the BDEs by considering their effect in both the halophosphine precursor (via so-called molecule stabilization enthalpies) as well as in the product phosphorus-centered radicals (via so-called radical stabilization enthalpies). Having investigated these effects, we note that the larger range of P–F BDEs, compared with the P–Cl BDEs, can be attributed to especially large stabilizing effects in F_2_P–F, (HO)_2_P–F, and to a lesser extent (H_2_N)_2_P–F, which were of noticeably greater magnitude than the corresponding effects in the chlorinated derivatives. Finally, to facilitate future studies concerning the strength of P–X (X = F or Cl) bonds in phosphorous (III) fluorides or chlorides toward homolytic bond dissociation in the gas phase, in molecules for which their size precludes being able to use a method such as W2, we examined the performance of a plethora of different DFT and DHDFT methods (in conjunction with the A′VQZ basis set) for their ability to compute P–F and P–Cl BDEs. As a result of this investigation, we identified DSD-PBEB95 as attaining the lowest mean absolute deviation (MAD) for both bond types (MADs = 2.1 and 2.2 kJ mol^−1^, respectively). Other recommended procedures for the computation of P–F BDEs are ωB97M-V (MAD = 2.2 kJ mol^−1^) and both M11 and M05-2X (both with MADs of 3.0 kJ mol^−1^). Concerning the computation of P–Cl BDEs, apart from DSD-PBEB95 which offered the best performance, we would also recommend BMK (MAD = 2.3 kJ mol^−1^) and either ωB97M-V, DSD-BLYP or DSD-PBEP86, which all attained MADs of 2.4 kJ mol^−1^.

## Data Availability

The datasets presented in this study can be found in online repositories. The names of the repository/repositories and accession number(s) can be found in the article/Supplementary Material.
