# Supplementary material for: The influence of substituents in governing the strength of the P–X bonds of substituted halophosphines R1R2P–X (X = F and Cl)
Source: Front Chem. 2023 Oct 3;11:1283418. doi: 10.3389/fchem.2023.1283418 (PMC10579588; doi:10.3389/fchem.2023.1283418)
Supplement: Supplementary file 1 [file DataSheet1.PDF]

Supporting Information for

The Influence of Substituents in Governing the Strength of the P–X Bonds of Substituted Halophosphines R1R2P–X (X = F and Cl)

Robert J. O'Reilly,* Amir Karton*

*E-Mails: roreill6@une.edu.au (RJO); amir.karton@une.edu.au (AK).

Table S1. Geometries of all molecules obtained at the B3LYP/A'VTZ level of theory

PH_2Cl

0 1

P	0.056995	1.034271	0.000000
Cl	0.056995	-1.054094	-0.000000
H	-0.911922	1.202771	1.022949
H	-0.911922	1.202771	-1.022949

PH_2F

0 1

P	0.074787	-0.545517	-0.000000
H	-0.897446	-0.738059	-1.022121
H	-0.897446	-0.738059	1.022121
F	0.074787	1.073208	0.000000

$H_2P•$

0 2

P	-0.000000	0.000000	0.116419
H	-0.000000	1.021491	-0.873142
H	-0.000000	-1.021491	-0.873142

PH_3

0 1

P	0.000000	-0.000000	0.127863
H	-0.000000	1.192286	-0.639313
H	1.032550	-0.596143	-0.639313
H	-1.032550	-0.596143	-0.639313

$PHCl_2$

0 1

P	0.028128	0.873098	-0.000000
Cl	0.028128	-0.416405	-1.627755
Cl	0.028128	-0.416405	1.627755
H	-1.378251	1.061314	-0.000000

PHFCl

0 1

P	-0.588349	0.588386	-0.111252
H	-0.647555	0.944166	1.266489
F	-1.464119	-0.747809	0.027721
Cl	1.332345	-0.178804	0.008988

ClHP•

0 2

P	0.042974	1.058123	0.000000
Cl	0.042974	-1.003689	0.000000
H	-1.375168	1.190868	-0.000000

PCl_3

0 1

P	-0.000000	0.000000	0.730976
Cl	0.000000	1.843503	-0.214993
Cl	1.596520	-0.921751	-0.214993
Cl	-1.596520	-0.921751	-0.214993

$PFCl_2$

0 1

P	-0.239490	0.711840	0.000000
Cl	-0.239490	-0.604062	1.596308
Cl	-0.239490	-0.604062	-1.596308
F	1.303890	1.095613	0.000000

$Cl_2P•$

0 2

P	0.000000	-0.000000	0.890584

Cl	-0.000000	1.607427	-0.392905
Cl	-0.000000	-1.607427	-0.392905

PF_3
0 1

P	0.000000	-0.000000	0.503130
F	-0.000000	1.373989	-0.279517
F	1.189910	-0.686995	-0.279517
F	-1.189910	-0.686995	-0.279517

PF_2Cl
0 1

P	-0.683024	0.209275	-0.000000
F	-0.683024	-0.837015	1.190460
F	-0.683024	-0.837015	-1.190460
Cl	1.325870	0.701597	0.000000

F_2P•
0 2

P	-0.000000	0.000000	0.566448
F	0.000000	1.210461	-0.472040
F	-0.000000	-1.210461	-0.472040

PHF_2
0 1

P	-0.041616	0.542083	0.000000
H	1.373334	0.752144	0.000000
F	-0.041616	-0.493521	1.216979
F	-0.041616	-0.493521	-1.216979

$(H_3C)HPCl$
0 1

P	-0.482488	0.715533	-0.109432
Cl	1.367182	-0.265030	0.009414
H	-0.537352	1.082368	1.260387
C	-1.635344	-0.721110	0.020922
H	-1.625999	-1.274498	-0.916681
H	-2.640179	-0.318461	0.169962
H	-1.389177	-1.390233	0.842245

$(H_3C)HPF$
0 1

P	-0.156973	-0.577080	-0.115267
F	-1.234363	0.630971	0.028414
H	-0.183679	-0.990974	1.247490
C	1.406713	0.382427	0.019457
H	1.598271	0.908718	-0.914747
H	2.220751	-0.328638	0.180058
H	1.388244	1.093798	0.843738

$(H_3C)HP$•
0 2

P	-0.056330	-0.707808	-0.000000
H	1.360325	-0.854108	-0.000000
C	-0.056330	1.147107	0.000000
H	-1.082614	1.510458	0.000000
H	0.452607	1.539059	-0.882338
H	0.452607	1.539059	0.882338

$(H_3C)PH_2$
0 1

P	0.070193	-0.673607	0.000000
H	-0.884504	-0.868947	-1.031076
H	-0.884504	-0.868947	1.031076
C	0.070193	1.189818	0.000000
H	0.610543	1.538772	-0.879428
H	0.610543	1.538772	0.879428
H	-0.926121	1.625549	-0.000000

$(H_2B)HPHCl$
0 1

P	0.561010	-0.595006	-0.143392
Cl	-1.336105	0.236417	0.034704
H	0.670913	-1.227445	1.114110
B	1.820843	0.768442	0.066155
H	1.608752	1.894311	-0.235071
H	2.914755	0.396930	0.351089

$(H_2B)HPF$
0 1

P	-0.031896	-0.489235	-0.140195
F	-1.270537	0.528023	0.068055
H	-0.040451	-1.149818	1.112367
B	1.556031	0.464421	0.059701
H	1.652062	1.634938	-0.104681
H	2.521507	-0.220909	0.184242

$(H_2B)HP$•
0 2

P	0.612825	-0.093373	0.036161
H	0.872291	1.205224	-0.484476
B	-1.266411	0.021191	-0.018074
H	-1.921877	-0.927694	-0.310300
H	-1.810734	1.017110	0.342737

$(H_2B)PH_2$

0 1

P	-0.060650	-0.559109	-0.000000
H	0.754167	-0.938295	1.083072
H	0.754167	-0.938295	-1.083072
B	-0.060650	1.299622	0.000000
H	-0.147665	1.882559	1.030906
H	-0.147665	1.882559	-1.030906

$(H_2N)HPCl$

0 1

P	0.546802	-0.639159	-0.098725
Cl	-1.403105	0.229488	-0.000749
H	0.549013	-0.975148	1.275598
N	1.649147	0.619083	0.034554
H	1.941409	1.054392	-0.826718
H	1.616311	1.273257	0.802859

$(H_2N)HPF$

0 1

P	-0.074708	-0.538628	-0.101049
F	-1.278405	0.562258	0.003220
H	-0.135636	-0.894304	1.272868
N	1.345007	0.335530	0.030290
H	1.790272	0.640396	-0.819906
H	1.556583	0.924288	0.821759

$(H_2N)HP$•

0 2

P	-0.643768	-0.098690	0.006116
H	-0.864861	1.305076	-0.025444
N	1.051446	0.017358	-0.038583
H	1.602596	-0.816172	0.077297
H	1.558664	0.869942	0.126487

$(H_2N)PH_2$

0 1

P	-0.598612	-0.124556	0.021903
H	-0.962376	0.537191	-1.189018
H	-0.953167	0.994663	0.817415
N	1.104224	0.040166	0.072239
H	1.613590	-0.800588	-0.146243
H	1.551567	0.855913	-0.316377

(HO)HPCl

0 1

P	-0.579173	-0.618391	-0.086312
Cl	1.358391	0.196911	-0.001437
O	-1.547082	0.693819	-0.081079
H	-0.587306	-0.922807	1.306568
H	-1.441091	1.300628	0.661170

(HO)HPF

0 1

P	-0.033413	0.523842	-0.130764
F	-1.249879	-0.524026	0.026995
O	1.267135	-0.431104	0.147378
H	-0.092333	0.993669	1.210371
H	1.705368	-0.686238	-0.670880

(HO)HP•

0 2

P	-0.020814	-0.607416	0.000000
O	-0.020814	1.052130	-0.000000
H	1.398173	-0.704294	0.000000
H	-0.919440	1.398502	-0.000000

$(HO)PH_2$

0 1

P	0.557660	-0.139566	0.000017
H	0.931791	0.771159	-1.024764
O	-1.082902	0.153520	-0.000018
H	0.931790	0.771414	1.024571
H	-1.565274	-0.677246	0.000080

FHP•

0 2

P	0.056859	-0.574457	0.000000
F	0.056859	1.038406	0.000000
H	-1.364616	-0.728805	0.000000

(HCO)HPCl

0 1

P	0.003534	0.807840	-0.134140
Cl	-1.701756	-0.371884	0.044634
C	1.322758	-0.539419	-0.087987
H	0.171329	1.101789	1.241175
O	2.473345	-0.260985	0.105138
H	0.982198	-1.572964	-0.301038

(HCO)HPF

0 1

P	0.553093	-0.575177	-0.119625
F	1.652145	0.598993	0.051965
C	-1.001059	0.470215	-0.016893
H	0.444784	-0.944247	1.249179
O	-2.094483	-0.026372	0.028644
H	-0.848276	1.570639	-0.050285

(HCO)HP•

0 2

P	-0.438407	0.993570	0.000000
C	0.000000	-0.802356	0.000000
H	-1.843924	0.764681	0.000000
O	1.155599	-1.164184	0.000000
H	-0.824762	-1.540617	0.000000

(HCO)PH$_2$

0 1

P	1.064705	-0.073751	-0.116763
H	1.531519	0.814614	0.881616
C	-0.725613	0.434779	0.012787
H	0.971604	-1.211299	0.715041
O	-1.653792	-0.329844	0.009008
H	-0.889681	1.533027	0.005998

(NC)HPCl

0 1

P	0.011413	0.904964	-0.107640
Cl	1.503216	-0.530105	0.010799
H	-0.008312	1.251451	1.266069
C	-1.446538	-0.126530	0.026072
N	-2.434047	-0.722135	0.001217

(NC)HPF

0 1

P	-0.602303	-0.568439	-0.113818
F	-1.374845	0.829008	0.031528
H	-0.656071	-0.962714	1.250103
C	1.096415	-0.000551	0.030119
N	2.212246	0.290219	-0.001043

(NC)HP•

0 2

P	0.000000	-1.063774	-0.000000
H	1.420291	-1.115150	0.000000
C	-0.038476	0.692667	0.000000
N	-0.169919	1.845108	0.000000

(NC)PH$_2$

0 1

P	0.120477	-1.027698	0.000000
H	-0.821746	-1.232864	-1.036819
H	-0.821746	-1.232864	1.036819
C	-0.027276	0.754837	-0.000000
N	0.000000	1.907453	-0.000000

(H$_3$Si)HPCl

0 1

P	0.141453	0.974718	-0.103635
Cl	1.630049	-0.504683	0.009481
H	0.099142	1.278980	1.282861
Si	-1.690000	-0.384061	0.006172
H	-1.797962	-1.105055	-1.279444
H	-2.861385	0.508463	0.178193
H	-1.612426	-1.346692	1.125324

(H$_3$Si)HPF

0 1

P	0.734954	0.607794	-0.109831
F	1.541111	-0.802967	0.028819
H	0.741313	0.969721	1.267929
Si	-1.425209	-0.146076	0.005905
H	-1.778447	-0.799933	-1.272938
H	-2.252764	1.071339	0.184152
H	-1.651497	-1.086265	1.126274

(H$_3$Si)HP•

0 2

P	0.042422	1.211258	0.000000
H	-1.380513	1.264535	-0.000000
Si	0.042422	-1.053236	-0.000000
H	1.442346	-1.533471	0.000000
H	-0.646031	-1.577314	-1.204632
H	-0.646031	-1.577314	1.204632

(H$_3$Si)PH$_2$

0 1

P	-0.053490	-1.173837	0.000000
H	0.909168	-1.285937	1.035887
H	0.909168	-1.285937	-1.035887
Si	-0.053490	1.097827	-0.000000
H	-0.783018	1.554158	1.204976
H	-0.783018	1.554158	-1.204976
H	1.298909	1.701541	-0.000000

$(H_2P)HPCl$

0 1

P	0.070883	0.901179	-0.101986
Cl	1.673122	-0.449128	0.005474
H	0.043083	1.215661	1.279915
P	-1.777768	-0.315607	0.035172
H	-1.590055	-0.981057	-1.199062
H	-1.292823	-1.382999	0.828301

$(H_2P)HPF$

0 1

P	0.660265	0.594939	-0.106557
F	1.536182	-0.766859	0.018582
H	0.685196	0.945660	1.271701
P	-1.447970	-0.065223	0.031121
H	-1.439765	-0.800187	-1.178024
H	-1.255491	-1.189491	0.870637

$(H_2P)HP$•

0 2

P	1.137169	-0.096844	0.021084
H	1.283944	1.312361	-0.108475
P	-1.045097	0.032384	-0.116086
H	-1.339487	-1.194348	0.524870
H	-1.325543	0.848883	1.008637

$(H_2P)PH_2$

0 1

P	0.000000	1.117947	-0.085622
H	0.189592	1.364402	1.296648
H	-1.409635	1.235542	-0.012323
P	-0.000000	-1.117947	-0.085622
H	1.409635	-1.235542	-0.012323
H	-0.189592	-1.364402	1.296648

(HS)HPCl

0 1

P	-0.011826	0.854073	-0.107976
Cl	-1.684950	-0.415085	0.004700
S	1.679436	-0.404935	0.093018
H	-0.000078	1.163736	1.273557
H	1.950645	-0.439432	-1.222106

(HS)HPF

0 1

P	-0.595656	0.580244	-0.116177
F	-1.542017	-0.725664	0.022134
S	1.363413	-0.181643	0.094192
H	-0.630957	0.937618	1.258628
H	1.629336	-0.204016	-1.222255

(HS)HP•

0 2

P	0.002543	1.087436	-0.000000
S	0.002543	-1.017502	0.000000
H	-1.418406	1.118667	-0.000000
H	1.339564	-1.150173	0.000000

$(HS)PH_2$

0 1

P	0.017023	1.063375	0.000000
H	-0.940940	1.244188	-1.028587
S	0.017023	-1.078330	-0.000000
H	-0.940940	1.244188	1.028587
H	1.354148	-1.185729	-0.000000

$(H_3C)_2PCl$

0 1

P	0.657577	-0.348927	-0.000000
Cl	-0.253726	1.549468	0.000000
C	-0.253726	-1.116871	-1.407905
C	-0.253726	-1.116871	1.407905
H	0.053868	-0.644857	2.339525
H	0.028230	-2.171496	1.450896
H	-1.334893	-1.035946	1.299387
H	0.053868	-0.644857	-2.339525
H	-1.334893	-1.035946	-1.299387
H	0.028230	-2.171496	-1.450896

$(H_3C)_2PF$

0 1

P	-0.337605	0.463716	0.000000
F	1.249827	0.836653	-0.000000
C	-0.337605	-0.726229	1.402253
C	-0.337605	-0.726229	-1.402253
H	-0.179219	-0.188806	-2.336677
H	-1.318080	-1.204355	-1.451884
H	0.430750	-1.492274	-1.289487
H	-0.179219	-0.188806	2.336677
H	0.430750	-1.492274	1.289487
H	-1.318080	-1.204355	1.451884

$(H_3C)2P$•

0 2

P -0.000000 0.000000 0.702026
C -1.407614 0.000000 -0.502230
C 1.407614 -0.000000 -0.502230
H 2.353962 -0.000000 0.036845
H 1.374375 0.883558 -1.144328
H 1.374375 -0.883558 -1.144328
H -2.353962 0.000000 0.036845
H -1.374375 -0.883558 -1.144328
H -1.374375 0.883558 -1.144328

$(H_3C)_2PH$
0 1
P 0.037694 -0.660891 0.000000
H -1.354846 -0.937345 0.000000
C 0.037694 0.525541 1.429398
C 0.037694 0.525541 -1.429398
H -0.156093 -0.023033 -2.351053
H 1.026973 0.975890 -1.515071
H -0.702324 1.319247 -1.327190
H -0.156093 -0.023033 2.351053
H -0.702324 1.319247 1.327190
H 1.026973 0.975890 1.515071

$(H_3C)PCl_2$
0 1
P 0.294651 0.697437 0.000000
Cl 0.294651 -0.618667 -1.619924
Cl 0.294651 -0.618667 1.619924
C -1.489318 1.138566 0.000000
H -1.685675 1.740900 -0.887906
H -1.685675 1.740900 0.887906
H -2.130633 0.259945 0.000000

$(H_3C)PClF$

0 1
P -0.417404 -0.159028 -0.579752
F -0.898396 -1.323681 0.421219
Cl 1.525389 0.130535 0.121661
C -1.230033 1.247927 0.264623
H -2.306456 1.159310 0.105908
H -0.885739 2.177930 -0.188094
H -1.012596 1.254654 1.331527

$(H_3C)ClP$•

0 2

P 0.000000 0.894526 0.000000
Cl -0.986756 -0.931219 0.000000
C 1.744101 0.302942 -0.000000
H 1.960226 -0.294168 0.886879
H 2.389786 1.183517 -0.000000
H 1.960226 -0.294168 -0.886879

$(H_3C)HPCl$
0 1
P 0.482488 0.715533 -0.109432
H 0.537353 1.082367 1.260388
Cl -1.367182 -0.265030 0.009414
C 1.635344 -0.721110 0.020922
H 2.640179 -0.318461 0.169961
H 1.626000 -1.274498 -0.916681
H 1.389177 -1.390234 0.842244

$(HO)_2PCl$
0 1
P 0.477845 -0.008200 0.558963
Cl -1.547269 -0.017017 -0.110950
O 1.065835 1.189367 -0.391860
O 1.096712 -1.248014 -0.254441
H 0.798357 2.071626 -0.109023
H 1.037153 -1.190172 -1.218871

$(HO)_2PF$
0 1
P 0.006534 -0.025490 0.514597
F 0.108172 -1.408201 -0.311355
O -1.225800 0.654090 -0.324839
O 1.212675 0.796752 -0.159455
H -2.089885 0.488628 0.066882
H 1.123319 0.960795 -1.109305

$(HO)_2P$•
0 2
P -0.008961 -0.603769 0.015199
O 1.192151 0.542063 -0.056077
O -1.286810 0.409010 -0.051584
H 2.045299 0.196937 0.230210
H -1.153612 1.251014 0.403098

$(HO)_2PH$
0 1
P 0.004360 0.543887 -0.100893
H 0.013264 0.933418 1.269544

O	-1.282258	-0.477253	0.122469
O	1.312785	-0.447414	-0.088912
H	-1.715786	-0.680460	-0.712231
H	1.392900	-1.013925	0.687625

$(H_2N)_2PCl$

0 1

P	0.448202	-0.031338	-0.578087
Cl	-1.580432	-0.123404	0.129019
N	1.345284	-1.103879	0.338035
N	0.851676	1.366304	0.342621
H	0.201681	2.129956	0.210115
H	1.797331	1.682044	0.159850
H	1.428469	-2.048894	-0.004208
H	1.338128	-1.032146	1.347636

$(H_2N)_2PF$

0 1

P	0.022063	-0.095660	-0.520570
F	-0.705242	-1.273261	0.338415
N	1.480916	0.189723	0.239749
N	-0.831733	1.176591	0.264867
H	-1.832999	1.123149	0.125895
H	-0.505076	2.093600	-0.013166
H	2.298499	-0.281520	-0.112375
H	1.511534	0.394824	1.230156

$(H_2N)_2P$•

0 2

P	-0.000000	0.000000	0.645181
N	0.000000	-1.270551	-0.500230
N	-0.000000	1.270551	-0.500230
H	0.131726	2.195705	-0.119413
H	-0.714537	1.258352	-1.217831
H	-0.131726	-2.195705	-0.119413
H	0.714537	-1.258352	-1.217831

$(H_2N)_2PH$

0 1

P	0.025921	-0.556696	0.000000
H	-1.369993	-0.780422	0.000000
N	0.025921	0.399968	1.415772
N	0.025921	0.399968	-1.415772
H	-0.617899	1.168422	-1.538359
H	0.927041	0.597233	-1.821610
H	0.927041	0.597233	1.821610
H	-0.617899	1.168422	1.538359

$(H_3Si)_2PCl$

0 1

P	-0.528270	-0.673974	0.000000
Cl	1.533428	-1.144826	-0.000000
Si	-0.528270	0.816551	-1.719807
Si	-0.528270	0.816551	1.719807
H	-0.244923	0.069360	2.963207
H	-1.895838	1.385473	1.776900
H	0.464427	1.899278	1.544723
H	-0.244923	0.069360	-2.963207
H	0.464427	1.899278	-1.544723
H	-1.895838	1.385473	-1.776900

$(H_3Si)_2PF$

0 1

P	-0.241903	0.847160	-0.000000
F	1.342634	1.282707	-0.000000
Si	-0.241903	-0.658929	1.715156
Si	-0.241903	-0.658929	-1.715156
H	-0.007006	0.073958	-2.978497
H	-1.603563	-1.243773	-1.724460
H	0.769633	-1.731062	-1.571773
H	-0.007006	0.073958	2.978497
H	0.769633	-1.731062	1.571773
H	-1.603563	-1.243773	1.724460

$(H_3Si)_2P$•

0 2

P	0.000000	1.089873	-0.000012
Si	-1.682565	-0.421954	0.000812
Si	1.682565	-0.421954	-0.000803
H	2.948958	0.263781	-0.342656
H	1.448739	-1.513733	-0.977293
H	1.808652	-1.016733	1.352033
H	-2.948949	0.263780	0.342699
H	-1.448718	-1.513741	0.977288
H	-1.808685	-1.016722	-1.352026

$(H_3Si)_2PH$

0 1

P	-0.024783	1.035384	0.000000
H	1.380059	1.238345	0.000000
Si	-0.024783	-0.438106	1.722223
Si	-0.024783	-0.438106	-1.722223
H	0.266299	0.306664	-2.968140
H	-1.382682	-1.021109	-1.812293

H	0.959194	-1.536614	-1.578515
H	0.266299	0.306664	2.968140
H	0.959194	-1.536614	1.578515
H	-1.382682	-1.021109	1.812293

$(H_2P)_2PCl$

0 1

P	0.020543	-0.081460	0.792790
Cl	1.039266	-1.605666	-0.244218
P	-1.977336	0.051238	-0.179851
P	0.891705	1.668575	-0.316723
H	2.073990	1.688428	0.467123
H	0.227545	2.605808	0.519806
H	-2.388404	-1.244971	0.212954
H	-1.604325	-0.328245	-1.491428

$(H_2P)_2PF$

0 1

P	0.008501	0.569517	0.615639
F	-0.012441	1.745846	-0.511947
P	1.640945	-0.781918	-0.065588
P	-1.646114	-0.690387	-0.236458
H	-2.662510	-0.077357	0.540933
H	-1.427494	-1.719886	0.715206
H	2.657920	0.188429	0.104775
H	1.494078	-0.561983	-1.457287

$(H_2P)_2P\bullet$

0 2

P	0.000000	-0.000000	1.012201
P	-0.000000	-1.651043	-0.451780
P	0.000000	1.651043	-0.451780
H	-0.667695	2.583358	0.379648
H	-1.158888	1.304518	-1.194455
H	0.667695	-2.583358	0.379648
H	1.158888	-1.304518	-1.194455

$(H_2P)_2PH$

0 1

P	0.000299	0.979780	-0.103265
H	0.014548	1.313054	1.275609
P	-1.673463	-0.512100	-0.077349
P	1.668124	-0.518890	0.088902
H	2.681074	0.475285	0.132450
H	1.804506	-0.701533	-1.311222
H	-2.699855	0.464034	-0.033190
H	-1.724667	-0.782677	1.312028

$(H_2N)PCl_2$

0 1

P	-0.018600	0.453006	0.622294
Cl	1.638264	-0.620631	-0.155264
Cl	-1.538177	-0.751149	-0.136812
N	-0.204537	1.755570	-0.404429
H	0.277943	2.599878	-0.131701
H	-0.268666	1.636295	-1.406402

$(H_2N)PClF$

0 1

P	0.470066	-0.080561	0.552807
F	0.774175	-1.354123	-0.370235
Cl	-1.524522	0.163825	-0.100549
N	1.304344	1.069182	-0.310560
H	1.653343	1.860487	0.207019
H	1.114565	1.265727	-1.283759

$(H_2N)PCl\bullet$

0 2

P	0.548864	-0.686106	0.022826
Cl	-1.356532	0.219389	-0.018188
N	1.607558	0.615989	0.028226
H	2.165660	0.799937	-0.793129
H	1.409519	1.450113	0.562365

$(H_2N)PClH$

0 1

P	0.546803	0.639159	-0.098725
H	0.549012	0.975145	1.275599
Cl	-1.403107	-0.229488	-0.000749
N	1.649147	-0.619083	0.034553
H	1.616316	-1.273259	0.802858
H	1.941412	-1.054389	-0.826718

$(H_2B)_2PCl$

0 1

P	-0.179023	0.471363	0.000000
B	0.080455	1.306640	1.601761
B	0.080455	1.306640	-1.601761
H	0.276438	0.702488	-2.598788
Cl	0.080455	-1.559752	-0.000000
H	0.276438	0.702488	2.598788
H	-0.019906	2.486983	-1.549119
H	-0.019906	2.486983	1.549119

$(H_2B)_2PF$
0 1

P	0.211130	-0.033141	-0.000000
B	-0.127575	-0.866370	1.582019
B	-0.127575	-0.866370	-1.582019
H	-0.458184	-0.270868	-2.549458
F	-0.127575	1.529622	0.000000
H	-0.458184	-0.270868	2.549458
H	0.086675	-2.032025	-1.565966
H	0.086675	-2.032025	1.565966

$(H_2B)_2P$•
0 2

P	-0.004160	0.641434	0.000000
B	-0.004160	-0.580751	1.405618
B	-0.004160	-0.580751	-1.405618
H	-0.822987	-0.525767	-2.264622
H	-0.822987	-0.525767	2.264622
H	0.874993	-1.381231	-1.456888
H	0.874993	-1.381231	1.456888

$(H_2B)_2PH$
0 1

P	0.000000	0.000000	0.375875
B	0.000000	1.628969	-0.451937
B	-0.000000	-1.628969	-0.451937
H	-0.000000	-2.624715	0.190347
H	0.000000	0.000000	1.774638
H	0.000000	2.624715	0.190347
H	-0.000000	-1.634809	-1.637044
H	0.000000	1.634809	-1.637044

$(HS)_2PCl$
0 1

P	0.055496	-0.020244	0.736518
Cl	1.910634	-0.275972	-0.247403
S	-1.239446	-1.386536	-0.261638
S	-0.777801	1.686421	-0.234127
H	-1.044079	-2.350324	0.653668
H	0.006811	2.547365	0.436664

$(HS)_2PF$
0 1

P	-0.229088	0.746716	-0.000000
F	1.314542	1.225628	-0.000000
S	-0.229088	-0.707080	1.557190
S	-0.229088	-0.707080	-1.557190
H	-0.531869	0.197588	2.503028
H	-0.531869	0.197588	-2.503028

$(HS)_2P$•
0 2

P	-0.000000	0.000000	0.956847
S	0.000000	1.570578	-0.472503
S	-0.000000	-1.570578	-0.472503
H	-0.506179	2.474617	0.383700
H	0.506179	-2.474617	0.383700

$(HS)_2PH$
0 1

P	0.022739	0.848840	0.000000
H	1.430484	0.968422	0.000000
S	0.022739	-0.419002	1.727772
S	0.022739	-0.419002	-1.727772
H	-1.249602	-0.146477	2.058114
H	-1.249602	-0.146477	-2.058114

$(NC)(H_2N)PCl$
0 1

P	0.014726	0.488185	0.614092
Cl	1.497030	-0.797841	-0.174962
C	-1.392200	-0.466963	0.027024
N	-0.146482	1.764213	-0.447732
N	-2.338105	-1.075411	-0.226900
H	0.401422	2.585842	-0.243720
H	-0.326509	1.634847	-1.433031

$(NC)(H_2N)PF$
0 1

P	0.478677	-0.083195	0.529847
F	0.801330	-1.344409	-0.428135
C	-1.256506	0.066848	0.031677
N	1.033721	1.250077	-0.287407
N	-2.391849	0.149183	-0.149898
H	1.923587	1.632246	-0.012198
H	0.730222	1.519451	-1.211214

$(NC)(H_2N)P$•
0 2

P	0.556296	-0.684055	0.000003
C	-1.071940	-0.006978	-0.000001
N	1.412055	0.768297	-0.000020
N	-2.173045	0.356075	-0.000000
H	2.417869	0.747325	0.000043

H 0.996267 1.684759 0.000061

$(NC)(H_2N)PH$
0 1
P 0.543011 0.629234 -0.100340
H 0.552639 1.063508 1.244541
C -1.151946 -0.003390 0.015325
N 1.527904 -0.736846 0.052862
N -2.238218 -0.392606 -0.015708
H 1.313325 -1.459703 0.723503
H 1.872751 -1.115828 -0.814977

$(NC)_2PCl$
0 1
P 0.769662 -0.041078 -0.000000
C -0.011492 0.863310 1.332241
C -0.011492 0.863310 -1.332241
Cl -0.367969 -1.759602 -0.000000
N -0.367969 1.440691 -2.264254
N -0.367969 1.440691 2.264254

$(NC)_2PF$
0 1
P 0.365515 0.690417 -0.000000
C 0.165865 -0.498576 -1.330105
C 0.165865 -0.498576 1.330105
F -1.088358 1.336201 -0.000000
N 0.165865 -1.171367 2.266177
N 0.165865 -1.171367 -2.266177

$(NC)_2P$•
0 2
P -0.000000 -0.000000 0.963832
C 1.318133 -0.000000 -0.204250
C -1.318133 0.000000 -0.204250
N -2.274495 0.000000 -0.857606
N 2.274495 -0.000000 -0.857606

$(NC)_2PH$
0 1
P 0.129766 0.925525 0.000000
C -0.027685 -0.226905 -1.355929
C -0.027685 -0.226905 1.355929
H -1.226682 1.326503 0.000000
N -0.027685 -0.891895 2.296971
N -0.027685 -0.891895 -2.296971

$(H_3C)PF_2$
0 1
P -0.538429 0.090962 -0.000000
F 0.282196 0.749860 -1.211897
F 0.282196 0.749860 1.211897
C 0.282196 -1.540409 0.000000
H -0.031390 -2.095708 -0.885106
H -0.031390 -2.095708 0.885106
H 1.366511 -1.428048 0.000000

$(H_3C)FP$•
0 2
P 0.000000 0.639952 -0.000000
F 1.338158 -0.275803 -0.000000
C -1.249307 -0.702163 0.000000
H -2.239884 -0.243059 0.000000
H -1.153851 -1.330507 0.887847
H -1.153851 -1.330507 -0.887847

$(H_2C{=}CH)HPCl$
0 1
P -0.018317 -0.831810 -0.047796
Cl -1.686809 0.446916 -0.090817
H -0.274201 -1.312162 1.262834
C 1.291219 0.341619 0.422598
C 2.420879 0.389553 -0.277566
H 1.142667 0.988820 1.279880
H 2.578960 -0.240469 -1.144835
H 3.230498 1.056363 -0.007235

$(H_2C{=}CH)HPF$
0 1
P -0.573170 -0.568784 0.021472
F -1.631410 0.633444 -0.256419
H -0.908591 -0.745584 1.394729
C 0.936853 0.393238 0.329047
C 2.086367 0.035335 -0.237139
H 0.893878 1.250263 0.993904
H 2.144674 -0.809702 -0.913222
H 3.010965 0.564362 -0.041182

$(H_2C{=}CH)HP$•
0 2
P 0.000000 -1.104902 -0.000000
H -1.339419 -1.581193 -0.000000
C -0.402776 0.625675 -0.000000
C 0.549504 1.583191 0.000000

H	-1.442920	0.931915	-0.000000
H	1.603999	1.333862	0.000000
H	0.297974	2.635749	0.000000

$(H_2C{=}CH)PH_2$

0 1

P	0.508669	-0.950561	0.000000
H	1.487886	-0.863353	1.025100
H	1.487886	-0.863353	-1.025100
C	0.000000	0.811426	0.000000
C	-1.283500	1.154864	0.000000
H	0.761005	1.584242	0.000000
H	-2.071004	0.410565	0.000000
H	-1.594808	2.192579	0.000000

$(HC{\equiv}C)HPCl$

0 1

P	0.045730	0.906266	-0.110617
C	-1.424721	-0.054552	0.037173
C	-2.478291	-0.638053	0.002579
H	-3.403996	-1.159877	-0.011179
Cl	1.531691	-0.562587	0.010438
H	0.097378	1.285498	1.254476

$(HC{\equiv}C)HPF$

0 1

P	-0.628558	-0.559976	-0.117116
C	1.051029	-0.035154	0.041061
C	2.222095	0.247372	0.001141
H	3.253232	0.504503	-0.015487
F	-1.412434	0.844130	0.031304
H	-0.751692	-0.975346	1.237273

$(HC{\equiv}C)HP$•

0 2

P	-0.037708	-1.099065	-0.000000
H	1.376349	-1.262560	0.000000
C	-0.000000	0.632376	0.000000
C	-0.104940	1.842078	-0.000000
H	-0.181093	2.901811	-0.000000

$(HC{\equiv}C)PH_2$

0 1

P	0.122378	-1.062264	0.000000
C	-0.039210	0.700511	-0.000000
C	0.000000	1.904056	-0.000000
H	0.015984	2.966160	-0.000000
H	-0.808199	-1.329803	-1.035507
H	-0.808199	-1.329803	1.035507

$(H_3C)(H_3Si)PCl$

0 1

P	-0.141231	0.439630	-0.706292
Cl	-1.542004	-0.863445	0.182293
Si	1.736179	-0.542910	0.139391
C	-0.261674	1.887609	0.447436
H	-1.220373	2.380454	0.291722
H	0.530004	2.589793	0.176307
H	-0.161217	1.616848	1.496821
H	1.882973	-1.880213	-0.472652
H	1.675988	-0.655252	1.615003
H	2.888707	0.307575	-0.247889

$(H_3C)(H_3Si)PF$

0 1

P	-0.543032	-0.156602	-0.594062
F	-0.988497	-1.348487	0.435931
Si	1.628107	0.103817	0.095240
C	-1.286718	1.289763	0.282948
H	-2.370317	1.252539	0.172147
H	-0.931314	2.200514	-0.204175
H	-1.032854	1.327651	1.341729
H	2.400897	-1.104493	-0.267991
H	1.730278	0.345629	1.554949
H	2.172073	1.271570	-0.640153

$(H_3C)(H_3Si)P$•

0 2

P	0.000000	1.010778	0.000000
Si	0.969114	-1.034329	0.000000
C	-1.781739	0.460290	0.000000
H	-2.426632	1.337545	0.000000
H	-2.011321	-0.139510	0.882622
H	-2.011321	-0.139510	-0.882622
H	2.441843	-0.881864	0.000000
H	0.565138	-1.809727	-1.201057
H	0.565138	-1.809727	1.201057

$(H_3C)(H_3Si)PH$

0 1

P	0.611039	0.741190	-0.107838
H	0.621883	1.123216	1.259097
Si	-1.436563	-0.222292	0.007097
C	1.703284	-0.772212	0.024556

H	2.731448	-0.445814	0.176783
H	1.663899	-1.312916	-0.920623
H	1.423616	-1.448375	0.830011
H	-2.449718	0.838619	0.212468
H	-1.556553	-1.225204	1.095007
H	-1.707977	-0.902004	-1.281870

$(O_2N)HPCl$

0 1

P	-0.262431	-0.933037	0.187933
Cl	-1.841375	0.343523	-0.073839
N	1.212435	0.221499	0.001714
H	-0.118028	-1.312090	-1.166957
O	1.123341	1.422140	0.091045
O	2.235510	-0.432482	-0.142142

$(O_2N)HPF$

0 1

P	0.815042	-0.652725	-0.146301
F	1.718862	0.633817	0.090786
N	-0.876996	0.108790	-0.003208
H	0.720776	-1.065585	1.208301
O	-1.045516	1.305956	-0.031217
O	-1.739133	-0.757134	0.055166

$(O_2N)HP\bullet$

0 2

P	-1.318060	-0.046481	-0.083001
N	0.535176	-0.000163	-0.001880
H	-1.395366	0.524109	1.214811
O	1.110474	-1.073148	0.042783
O	1.067030	1.094929	-0.037362

$(O_2N)PH_2$

0 1

P	0.053735	-1.284004	-0.000000
H	-0.895312	-1.398417	1.040089
N	0.017835	0.575133	0.000000
H	-0.895312	-1.398417	-1.040089
O	0.053735	1.126935	1.085353
O	0.053735	1.126935	-1.085353
